# A Network Model to Describe the Terminal Differentiation of B Cells

**DOI:** 10.1371/journal.pcbi.1004696

**Published:** 2016-01-11

**Authors:** Akram Méndez, Luis Mendoza

**Affiliations:** 1 Programa de Doctorado en Ciencias Bioquímicas, Universidad Nacional Autónoma de México, Ciudad de México, México; 2 Instituto de Investigaciones Biomédicas, Universidad Nacional Autónoma de México, Ciudad de México, México; 3 C3, Centro de Ciencias de la Complejidad, Universidad Nacional Autónoma de México, Ciudad de México, México; German Cancer Research Center, GERMANY

## Abstract

Terminal differentiation of B cells is an essential process for the humoral immune response in vertebrates and is achieved by the concerted action of several transcription factors in response to antigen recognition and extracellular signals provided by T-helper cells. While there is a wealth of experimental data regarding the molecular and cellular signals involved in this process, there is no general consensus regarding the structure and dynamical properties of the underlying regulatory network controlling this process. We developed a dynamical model of the regulatory network controlling terminal differentiation of B cells. The structure of the network was inferred from experimental data available in the literature, and its dynamical behavior was analyzed by modeling the network both as a discrete and a continuous dynamical systems. The steady states of these models are consistent with the patterns of activation reported for the Naive, GC, Mem, and PC cell types. Moreover, the models are able to describe the patterns of differentiation from the precursor Naive to any of the GC, Mem, or PC cell types in response to a specific set of extracellular signals. We simulated all possible single loss- and gain-of-function mutants, corroborating the importance of Pax5, Bcl6, Bach2, Irf4, and Blimp1 as key regulators of B cell differentiation process. The model is able to represent the directional nature of terminal B cell differentiation and qualitatively describes key differentiation events from a precursor cell to terminally differentiated B cells.

## Author Summary

Generation of antibody-producing cells through terminal B cell differentiation represents a good model to study the formation of multiple effector cells from a progenitor cell type. This process is controlled by the action of several molecules that maintain cell type specific programs in response to cytokines, antigen recognition and the direct contact with T helper cells, forming a complex regulatory network. While there is a large body of experimental data regarding some of the key molecules involved in this process and there have been several efforts to reconstruct the underlying regulatory network, a general consensus about the structure and dynamical behavior of this network is lacking. Moreover, it is not well understood how this network controls the establishment of specific B cell expression patterns and how it responds to specific external signals. We present a model of the regulatory network controlling terminal B cell differentiation and analyze its dynamical behavior under normal and mutant conditions. The model recovers the patterns of differentiation of B cells and describes a large set of gain- and loss-of-function mutants. This model provides an unified framework to generate qualitative descriptions to interpret the role of intra- and extracellular regulators of B cell differentiation.

## Introduction

**Fig 1:**
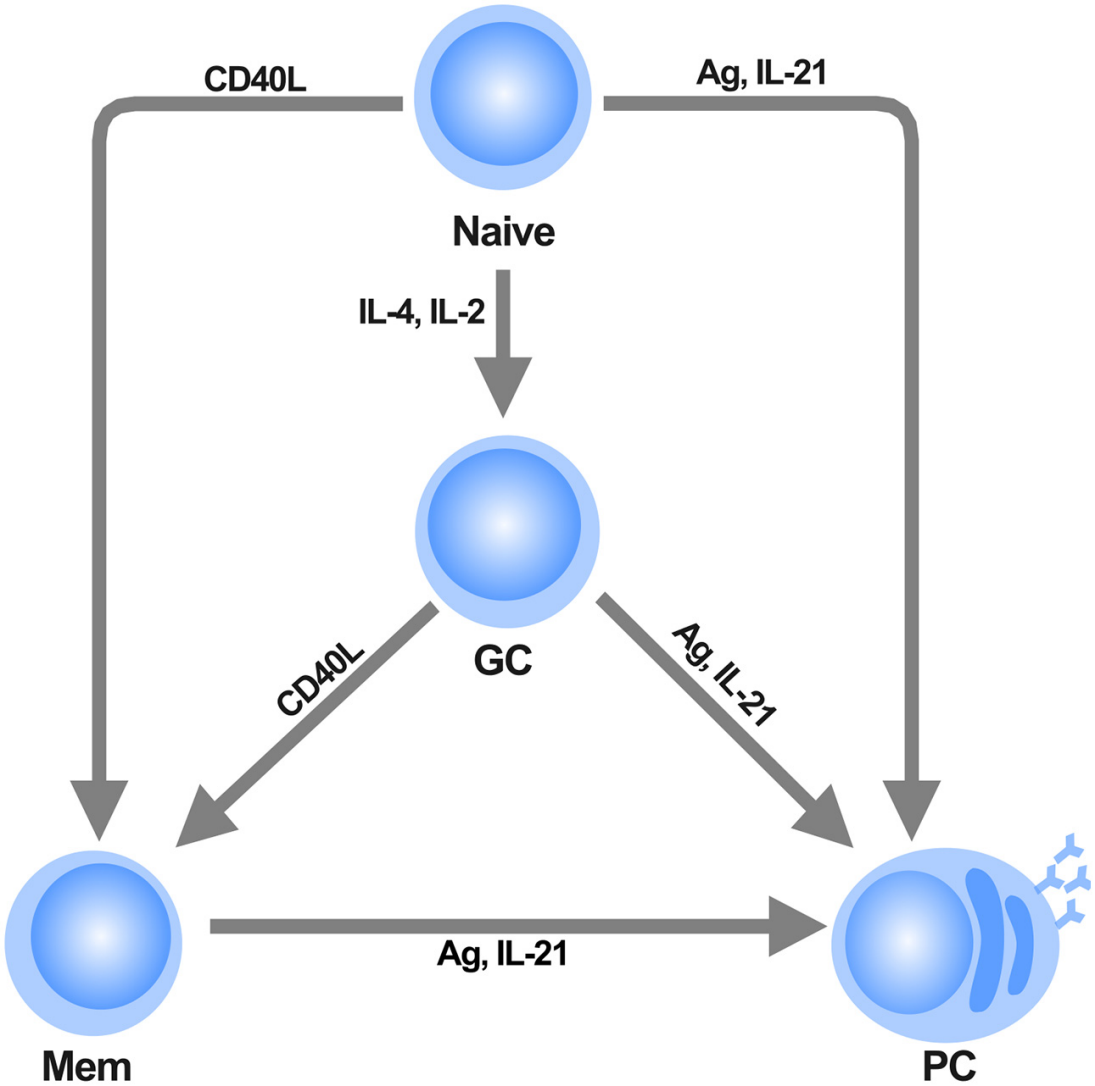
Terminal B cell differentiation. Precursor Naive B cells can differentiate into three possible cell types depending on proper molecular stimuli. Cytokines secreted by T-helper cells play a central role in the determination of B cell fate. IL-2 and IL-4 are required for the transition of Naive to GC cells. Direct contact of B cells with T cells by means of the CD40L receptor promote the differentiation of Naive or GC cells toward the Mem cell type. Antigen (Ag) activation drives terminal differentiation toward the PC cells, a process that is favored by the presence of IL-21.

Adaptive immunity in vertebrates depends on the rapid maturation and differentiation of T and B cells. While T cells originate cell-mediated immune responses, B cells are responsible for the humoral response of the organism by means of the production of high-affinity antibodies. B cells develop in the bone marrow from hematopoietic progenitors, and migrate as mature B cells (Naive) to the germinal centers (GCs), which are highly specialized environments of the secondary lymphoid organs [1]. There, B cells are activated by antigens (Ag) and undergo diversification of the B cell receptor (BCR) genes by somatic hypermutation (SHM), as well as the subsequent expression of distinct isotypes by class switch recombination (CSR) [2]. After the activation due to Ag recognition, Naive and GC B cells differentiate into antibody-producing plasma cells (PC), as well as memory cells (Mem) [3]. Cytokines secreted by T-helper cells, such as IL-2, IL-4 and IL-21 as well as the direct contact with these cells, mediated by the union CD40 receptor on B cells with its ligand CD40L, play a key role in the determination of B cell fate [4], since these external signals act as instructive cues that promote the differentiation from a cell progenitor to multiple cell types (Fig 1).

Terminal differentiation of B cells is controlled by the concerted action of multiple transcription factors that integrate physiologic signals in response to BCR cross-linking, extracellular cytokines, and the direct interaction with T cells, thus creating a complex regulatory network. These factors appear to regulate mutually antagonistic programs and can be divided into those that promote and maintain B cell identity, such as Pax5, Bcl6 and Bach2, and those that control differentiation into memory cells or plasma cells, i.e., Irf4, Blimp1 and XBP1, as has been shown by multiple functional, biochemical and gene expression analysis [5–7].

A type is characterized by the expression of a specific set of master transcriptional regulators. Naive B cells express Pax5 and Bach2, which are induced at the onset of B cell development, and are maintained through all developmental stages upon plasma cell differentiation [8, 9]. Furthermore, Pax5 is essential for the maintenance of B cell identity, since Pax5 deficiency results in the acquisition of multilineage potential [10]. Both Pax5 and Bach2 are required to inhibit PC differentiation [11, 12]. In addition to Pax5 and Bach2, GC cells express Bcl6, a transcription factor necessary for germinal center formation that allows the SHM and CSR processes to occur [13–15]. Development of B cells toward Mem cells requires Bcl6 downregulation and the induction of Irf4 [16, 17]. Conversely, PCs are characterized by the expression of Blimp1 and XBP1 that along with Irf4, inhibit the B cell identity program [5, 18].

Although a number of molecules that play a key role in the process of the terminal differentiation of B cells are known, it is not completely clear how such molecules regulate each other to ensure the proper appearance of GC, Mem, and PC from progenitor Naive B cells. There exist models describing several aspects of the differentiation of B cells such as the decisions promoting the developmental processes of CSR and SHM [19, 20], the response to environmental contaminants that disrupt B cell differentiation [21, 22], the B cell exit from the GC phase for the differentiation into plasma or memory cells [23], as well as the dynamics of B cell differentiation inside the complex microenvironment of germinal centers [24, 25]. Nonetheless, a general consensus about the regulatory network controlling cell fate decisions of B lymphocytes is lacking.

The modeling of regulatory networks has been shown to be a valuable approach to understand the way cells integrate several signals that control the differentiation process [26, 27]. In particular, the logical modeling approach has been useful to qualitatively describe biological processes for which detailed kinetic information is lacking [28]. This type of modeling usually focus on the nature and number of steady states reached by the network, which are often interpreted as stable patterns of gene expression that characterize multiple cell fates [29]. In this paradigm, the transit from one steady state to another occurs when cells receive a specific external stimuli, such as hormones, cytokines, changes in osmolarity, etc. These external stimuli are sensed and integrated to create an intracellular response that may trigger a global response such as cell growth, division, differentiation, etc. External signals are usually continuous in nature, i.e., they are present as concentration gradients of external molecules that may attain different values of strength and duration. Therefore it becomes desirable to develop models that incorporate the possibility of following the response of the network to continuous signals while at the same time, describe qualitatively the directional and branched nature of cell differentiation processes.

In this work we infer the regulatory network that controls the terminal differentiation of B cells. We then construct two dynamical systems, one discrete and one continuous, to analyze the dynamical properties of the regulatory network. Specifically, we find the stationary states of the models, and compare them against the known stationary molecular patterns observed in Naive, GC, Mem, and PC cells, under wild type and mutant backgrounds. Finally, we show that the dynamical models are able to describe the cellular differentiation pattern under a variety of external signals. Importantly, the models predict the existence of several interactions necessary for the network to ensure the proper pattern of terminal differentiation of B cells. Furthermore, the continuous model predicts the existence of intermediary states that could be reached by the network, but that have not been reported experimentally.

## Results

**Fig 2:**
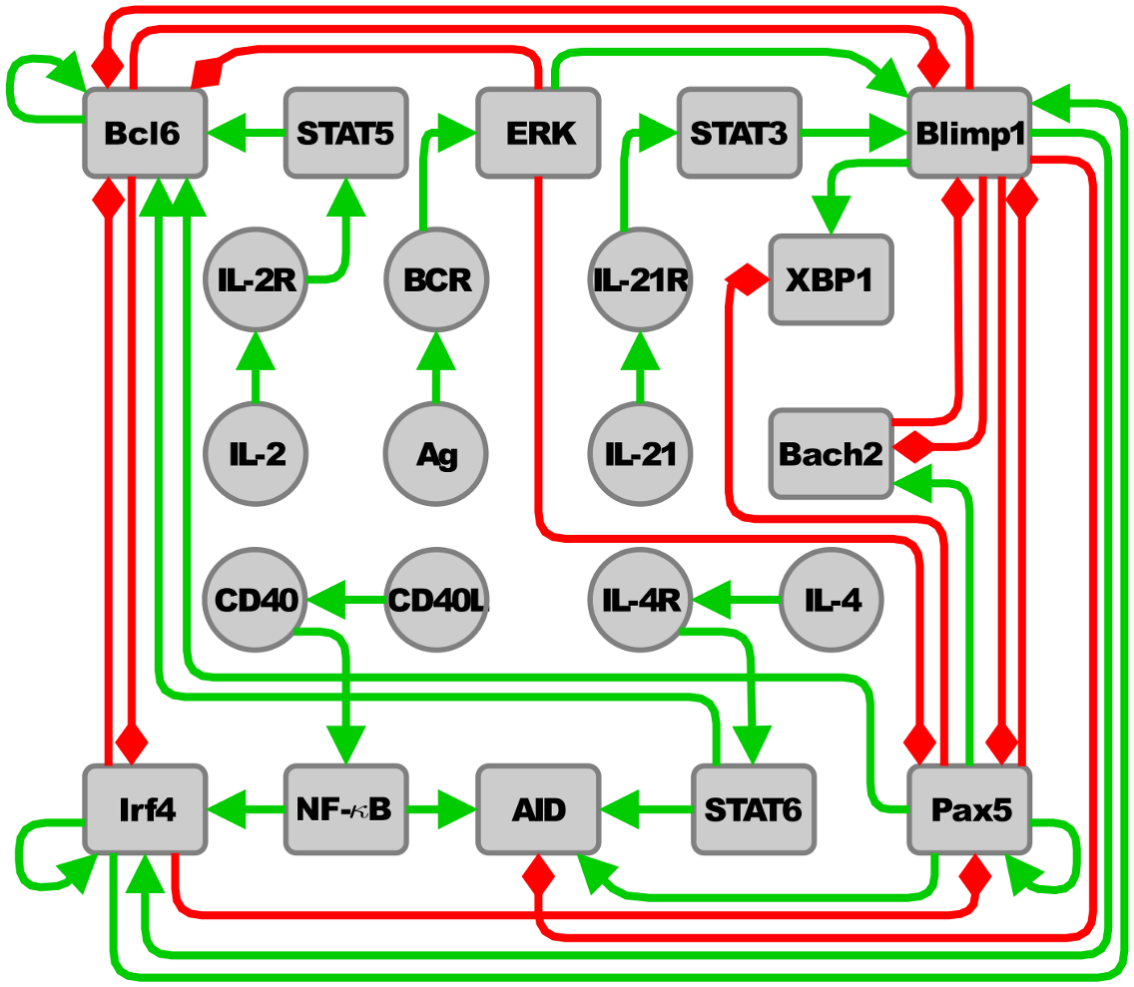
The regulatory network of B cells. Nodes represent molecules or molecular complexes. Positive and negative regulatory interactions among molecules are represented as green continuous arrows and red blunt arrows respectively.

We inferred the regulatory network that controls the terminal differentiation of B cells from experimental data available in the literature referring to the key molecules involved in the control of terminal B cell differentiation from the precursor B cell (Naive) to GC, Mem or PC cell types (Fig 2). The network contains 22 nodes representing functional molecules or molecular complexes, namely AID, Ag, Bach2, Bcl6, BCR, Blimp1, CD40, CD40L, ERK, IL-2, IL-2R, IL-4, IL-4R, IL-21, IL-21R, Irf4, NF-*κ*B, Pax5, STAT3, STAT5, STAT6 and XBP1. These nodes have 39 regulatory interactions among them, being either positive or negative. S1 Table contains the set of key references used to infer the regulatory network depicted in Fig 2.

The regulatory network consists of two sets of nodes, i.e., those pertaining to a core module integrated by the master transcriptional regulators of terminal B cell differentiation (Bach2, Bcl6, Blimp1, Irf4, Pax5 and XBP1) and a set of nodes representing several signal transduction cascades (Ag/BCR/ERK, CD40L/CD40NF-*κ*B, IL-2/IL-2R/STAT5, IL-4/IL-4R/STAT6 and IL-21/IL21R/STAT3) representing key external signals required for the control of the differentiation process. The nodes corresponding to these signaling pathways are active if a external stimuli is present, i.e., if a extracellular molecule is available, it is recognized by a specific receptor that transduce the signal by a messenger molecule, which in turn regulates the expression of the transcription factors in the core regulatory network. Most interactions among the nodes in Fig 2 were inferred from the literature. However, we found necessary to incorporate to the network the interactions Pax5 → Bcl6, Irf4 ⊣ Pax5 and the self-regulatory interactions Bcl6 → Bcl6, and Pax5 → Pax5 so as to obtain attractors with biological significance. Therefore, these four regulatory interactions constitute predictions of our model. The following paragraphs resume the reasons to incorporate such unreported interactions into our regulatory network model.

B cells develop in the bone marrow from hematopoietic progenitors that progressively lose its multipotent potential as they commit with the B cell lineage. This process strictly depends on the expression of Pax5, which induces chromatin changes of B cell specific genes and restricts the developmental potential of lymphoid progenitors by repressing genes associated with other cell type programs [30, 31]. Pax5 is upregulated at the onset of B cell development until differentiation to plasma cells [7]. During early stages of B cell development, Pax5 expression is positively controlled by the transcription factor Ebf1 [32], which in turn is activated by Pax5 [33], thus conforming a mutually activatory regulatory circuit that controls B cell identity. However, the signals that maintain Pax5 expression throughout late stages of B cell differentiation are not well understood. Therefore, a positive autoregulatory interaction for Pax5 was included in order to account for the direct mechanisms, possibly via the positive regulatory circuit between Ebf1 and Pax5, or indirect mechanisms, via other signals, that might sustain high Pax5 expression during late B cell differentiation.

Once B cells have completed their development in the bone marrow, they migrate to the bloodstream into the secondary lymphoid organs where they complete maturation throughout the germinal center reaction. The transcription factor Bcl6 is essential for germinal center formation, since Bcl6 deficiency results in the absence of germinal centers in mice [9, 15]. Given that Pax5 is required from the beginning of B cell development [10], it was necessary to include a positive regulatory signal from Pax5 to Bcl6 to keep Bcl6 in an active state when the Pax5 node is active.

A high expression of Bcl6 is required during the GC phase where it controls the expression of genes necessary for the germinal center program, such as DNA damage response and apoptosis, thus promoting the processes of SHM and CSR and cell proliferation [6]. It has been shown that mutations that disrupt a negative autoregulatory circuit deregulate Bcl6 expression and contribute to extensive proliferation in dense large B cell lymphoma (DLBCL) [34]. Moreover, it has been reported that in normal conditions there exist epigenetic mechanisms associated with positive regulation of Bcl6 expression during the GC phase that overcome its negative autoregulation [35, 36]. However, the precise mechanisms and signals that maintain high levels of Bcl6 in GCs are not fully understood. Therefore, we found necessary to include a positive autoregulatory interaction for Bcl6 in order to account for the possible role of these mechanisms in GC cell differentiation.

Direct contact of B cells with T cells mediated by the union of the CD40 receptor with its ligand CD40L induce the expression of Irf4 [16]. It has been shown that low levels of Irf4 promote the early B cell program, while high Irf4 levels inhibit GC program and promote differentiation toward the Mem or PCs in later stages of B cell differentiation [18, 37]. Given that Pax5 is an essential regulator of the B cell identity program [10], a negative interaction between Pax5 and Irf4 was incorporated to simulate a constant activation of the Pax5 circuit when the Irf4 node is low and to inhibit Pax5 activation when Irf4 present at high levels.

### Attractors of the wild type network

We studied the dynamical behavior of the discrete and the continuous systems so as to obtain their attractors. The discrete version of the B cell regulatory network was studied by exhaustively testing the behavior of the network from all possible initial conditions. The system reaches exactly four fixed point attractors, shown in Table 1. Notably, there is a one-to-one relation of these four attractors with the expression patterns of the cell types shown in Fig 1.

**Table 1:** Attractors of the discrete and continuous models of the B cell regulatory network.

	Naive		GC		Mem		PC	
Node	Disc.	Cont.	Disc.	Cont.	Disc.	Cont.	Disc.	Cont.
Bach2	1	1.0*E* + 0	1	1.0*E* + 0	1	1.0*E* + 0	0	1.1*E* − 22
Bcl6	0	1.8*E* − 22	1	1.0*E* + 0	0	1.4*E* − 22	0	1.0*E* − 22
Blimp1	0	2.3*E* − 22	0	1.3*E* − 22	0	1.3*E* − 22	1	1.0*E* + 0
Irf4	0	1.7*E* − 22	0	9.7*E* − 23	1	1.0*E* + 0	1	1.0*E* + 0
Pax5	1	1.0*E* + 0	1	1.0*E* + 0	1	1.0*E* + 0	0	1.0*E* − 22
XBP1	0	2.3*E* − 22	0	1.2*E* − 22	0	9.9*E* − 23	1	1.0*E* + 0

For the continuous system we present averages from a total of 500,000 runs from random initial states. The standard deviation are smaller than 1*E*^−22^ in all cases. For simplicity, only the nodes conforming the network core are shown. The rest of nodes belong to the signal transduction cascades, and all of them are in the inactive state, *i.e.* 0.

**Fig 3:**
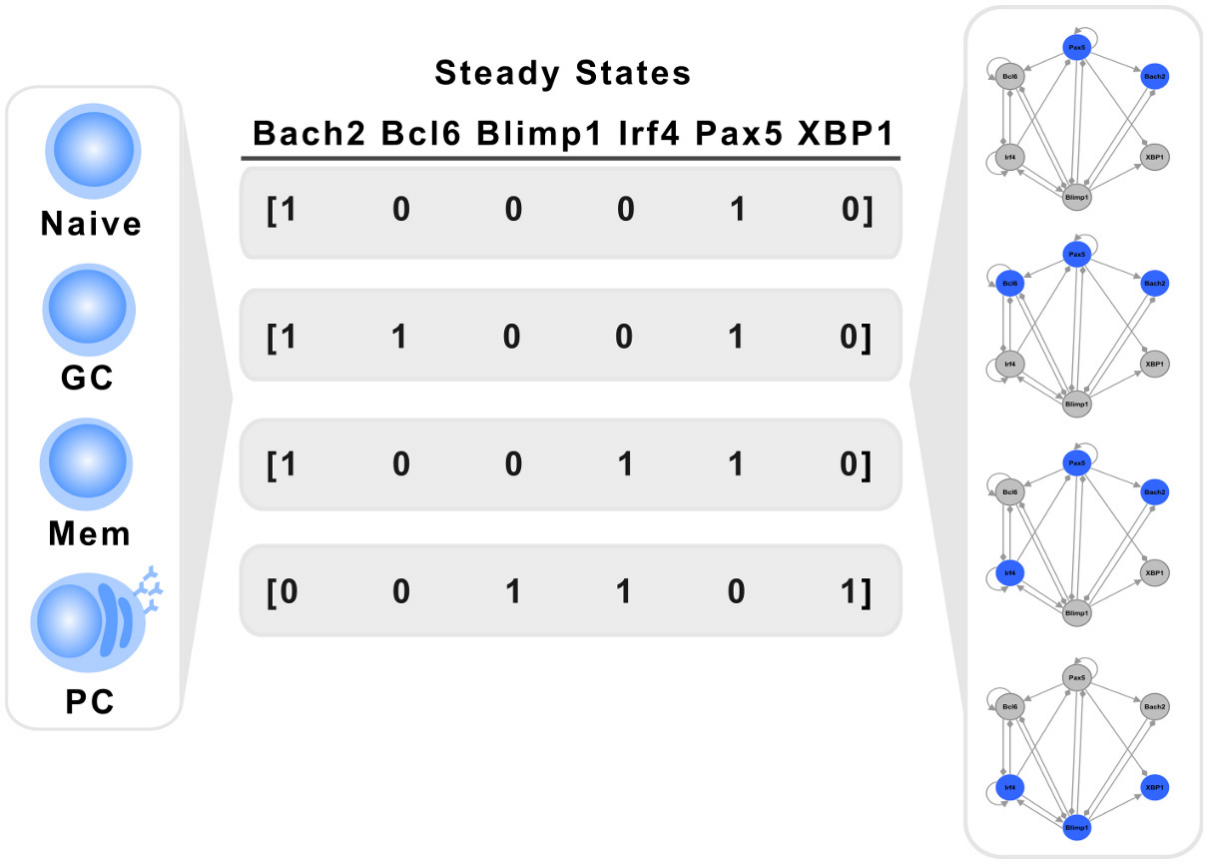
Attractors and cell types The stationary states of the regulatory network correspond to multiple activation patterns that characterize different cell types.

We labeled the attractors as Naive, GC, Mem, and PC. It is important to remember that each attractor represents a different configuration pattern of the network at the steady state. Specifically, the first attractor, where the nodes Pax5 and Bach2 are active, can be interpreted as the activation pattern of Naive cells. The second attractor, with high levels of Bcl6, Pax5, and Bach2, corresponds to the GC cell type. The third attractor, with high levels of Irf4, Pax5, and Bach2, along with the absence of Bcl6 can be interpreted as the Mem cell fate. Finally, the fourth attractor, with high Blimp1, Irf4, and XBP1, corresponds to the pattern of the cell type PC (Fig 3).

In the discrete version of the model, the set of initial states draining to the attractors, i.e the basins of attraction, do not partition the state space evenly. The percentage of initial states leading to each of the attractors were as follows: Naive = 56.25%, GC = 6.25%, Mem = 6.25%, PC = 31.25%. The size of the basins reflects how an attractor can be attained from different initial configurations, and may indicate the relative stability of such steady state [38]. It has been suggested that different basins represent stable or semistable cellular differentiation states [29]. Moreover, in order to transit form one steady state to another, a specific external signal would need to trigger a response in order to overcome the basin of attraction such that a different attractor could be reached by the system. Configurations with larger basins can be easily reached from many initial states, therefore, different perturbations could be buffered and canalized by the network towards a particular steady state [39].

Since the Naive and PC states have larger basins than that for GC or Mem attractors, it is possible to suggest that the former are relatively more stable than the later. Importantly, the proportion of basin sizes of the Naive, GC and Mem attractors agree with *in vivo* measures of B cells where the Naive progenitor is more abundant in proportion than the other three cell types [40, 41]. However in spite of the low abundance of PC cells *in vivo* as a result of a selection process of B lymphocytes during the germinal center reaction, the largest basins corresponding to the progenitor Naive and terminally differentiated PC cells suggest that the regulatory network assures the formation of these cell types in a robust manner.

Contrary to discrete systems, continuous dynamical systems have an infinite number of possible initial states so that the search for attractors by sampling a large number of random initial states can lead to the possibility to miss attractors with small basins of attraction. Indeed, the sampling of initial states resulted in the finding of only four attractors for the continuous model, which resulted identical to the attractors of the discrete model, see Table 1. Therefore, to find possible missing attractors we made an exhaustive perturbation study by temporarily modifying the activation state of each node in the four attractors found by random sampling [42]. With this approach we found three more fixed point attractors in the continuous model. These extra attractors are characterized by intermediate values of activation of the nodes conforming the network core and do not have a counterpart in the discrete model, since the discrete model can attain only 0 or 1 activation values (Table 2).

**Table 2:** Fixed point attractors of the continuous system not found in the random search.

	Attractor		
Node	New1	New2	New3
AID	0	0	0
Ag	0	0	0
Bach2	1	1	1
Bcl6	0.5	0	0.5
BCR	0	0	0
Blimp1	0	0	0
CD40	0	0	0
CD40L	0	0	0
ERK	0	0	0
IL-2	0	0	0
IL-2R	0	0	0
IL-4	0	0	0
IL-4R	0	0	0
IL-21	0	0	0
IL-21R	0	0	0
Irf4	0	0.5	0.5
NF-*κ*B	0	0	0
Pax5	1	1	1
STAT3	0	0	0
STAT5	0	0	0
STAT6	0	0	0
XBP1	0	0	0

Three fixed point attractors were found with the perturbation analysis that has not been found in the random search. These attractors are characterized by intermediate values of the nodes.

These attractors with intermediate values may represent possible unstable activation states that can be reached by the system but have not been yet experimentally observed or may correspond to transient differentiation states. Indeed, one of the attractors (“New3” attractor) found in Table 2 shows intermediate levels of Bcl6 and Irf4, in spite of the antagonistic role of these two factors, suggesting that low levels of Irf4 controls the establishment of stationary states prior to Bcl6 downregulation. This attractor may correspond to the known activation pattern of centrocytes, which are Irf4^int^, Bcl6^hi^ B cells exiting the GC reaction that represent an intermediate cellular state between GC and PC cells [43]. This result supports the role of Irf4 as a regulator of the differentiation process prior the terminal differentiation to PCs since it has been observed that intermediate levels of Irf4 promote the GC program, whereas high levels of Irf4 promote Bcl6 downregulation and further PC differentiation as B cells exit the germinal center [44].

### The differentiation process

**Fig 4:**
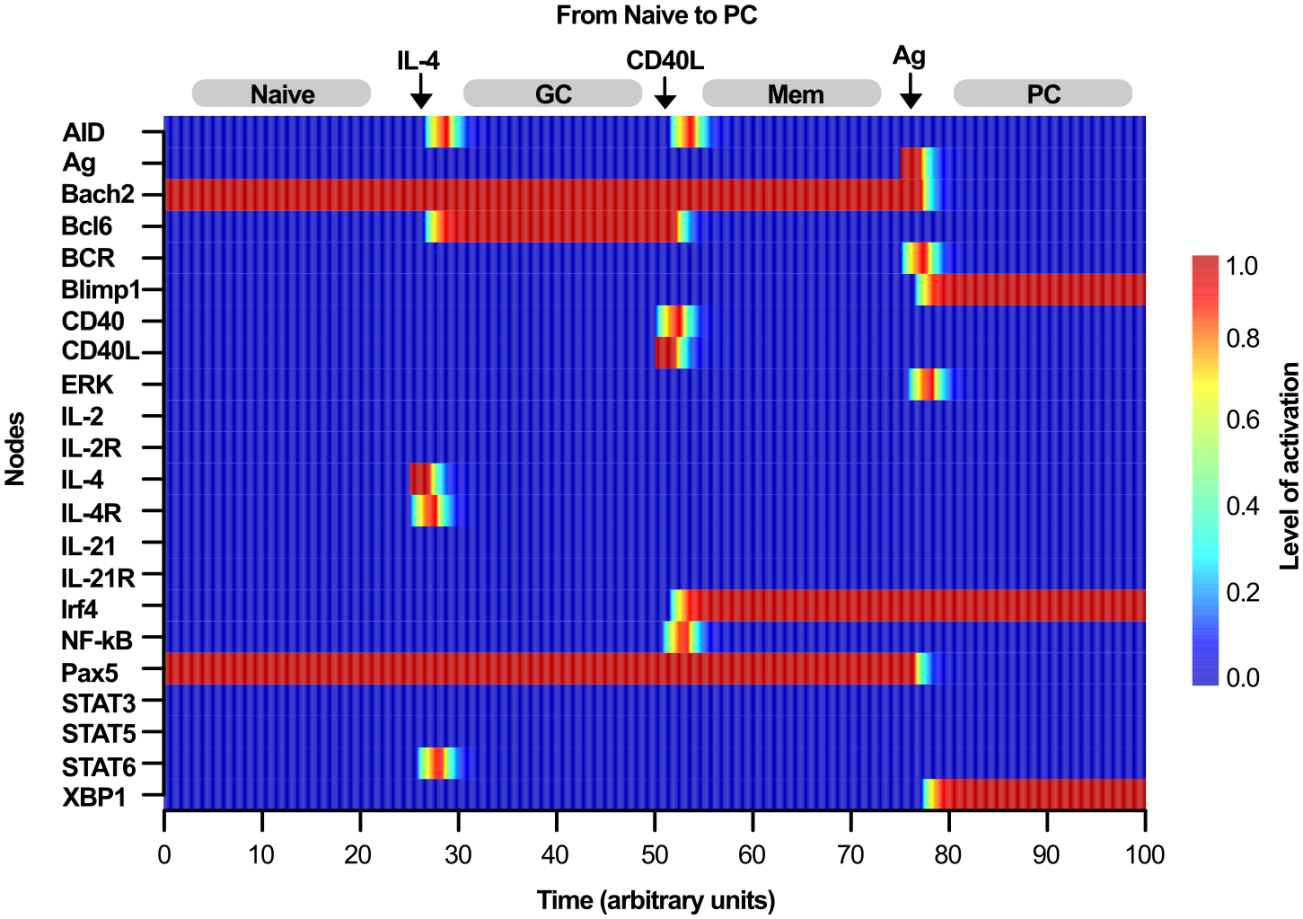
Differentiation from Naive to the PC cell type. The changes in the activation of all nodes of the network are shown as a heatmap which scales from blue to red as the activation level goes from 0 to 1, respectively. Extracellular signals are simulated as a burst for two or more units of time (arrows). Starting from the Naive (Bach2^+^, Pax5^+^) stationary state (*t* = 0 to *t* ≈ 25), the system moves to the GC attractor (Bach2^+^, Bcl6^+^, Pax5^+^) due to the presence of a simulated pulse of IL-4 (*t* ≈ 25) which in turn transit to the Mem attractor (Bach2^+^, Irf4^+^, Pax5^+^) due to the action of CD40L (*t* ≈ 55) and finally, Mem attractor moves to the PC state (Blimp1^+^, Irf4^+^) by the presence of Ag signal (*t* ≈ 75).

The B cell regulatory network is able to describe the differentiation process outlined in Fig 1, from the Naive precursor to any of the GC, Mem, or PC cell types by means of sequential pulses of extracellular signals known to direct terminal B cell differentiation (Fig 4). The system is initialized starting from the Naive attractor, and the system is perturbed at a time *t* ≈ 25 with a single high pulse of IL-2 or IL-4 for 2 or more time units. Computationally, this is achieved by fixing the variable *IL* − 4 = 1 and the equation $\frac{dIL-4}{dt}=0$ for the indicated period of time. This signal was intended to mimic the effect of subjecting the Naive cell to a saturating extracellular concentration of IL-2 or IL-4 for a brief incubation time. After the pulse, the entire system was left to evolve until it converged. This perturbation is sufficient to move the dynamical system to the GC attractor which is in agreement with the observations that IL-2 and IL-4 promote B cell proliferation and germinal center formation, and are also necessary signals for the transition of Naive B cells to GC B cells [45–47].

Differentiation of either Naive or GC cells to Mem cells is mediated by the activation of the CD40 receptor by its ligand CD40L [48], which leads to Irf4 induction and to the repression of Bcl6 [16]. Our model recovers these differentiation routes with a saturating activation of CD40L for ≈2 or more abitrary time units, which leads to the activation of Irf4 node when the Pax5 node is active and Blimp1 is not present. Activation of Irf4 downregulates Bcl6 and directs the transition from the GC to the Mem attractor of the dynamical system, see Fig 4.

Similarly, starting from any of the Naive, GC, or Mem attractors, the system is able to move to the PC attractor by applying a saturating signal of either IL-21 or Ag. This is consistent with the experimental reports where BCR activation by Ag induce Blimp1 upregulation, as well as Pax5 and Bcl6 downregulation thus promoting plasma cell differentiation from either Naive, GC, or Mem cell types [49–51]. This process is facilitated by the presence of IL-21 which is transduced by STAT3 [52, 53].

For both the discrete and continuous models we obtained the same biological relevant transition paths that describe the wild type differentiation pattern outlined in Fig 1. However, given that the continuous model has 7 fixed-point attractors, its complete fate map is larger than that for the discrete model (S1 Fig). Nonetheless, the continuous model also presents the known biologically relevant transitions.

**Fig 5:**
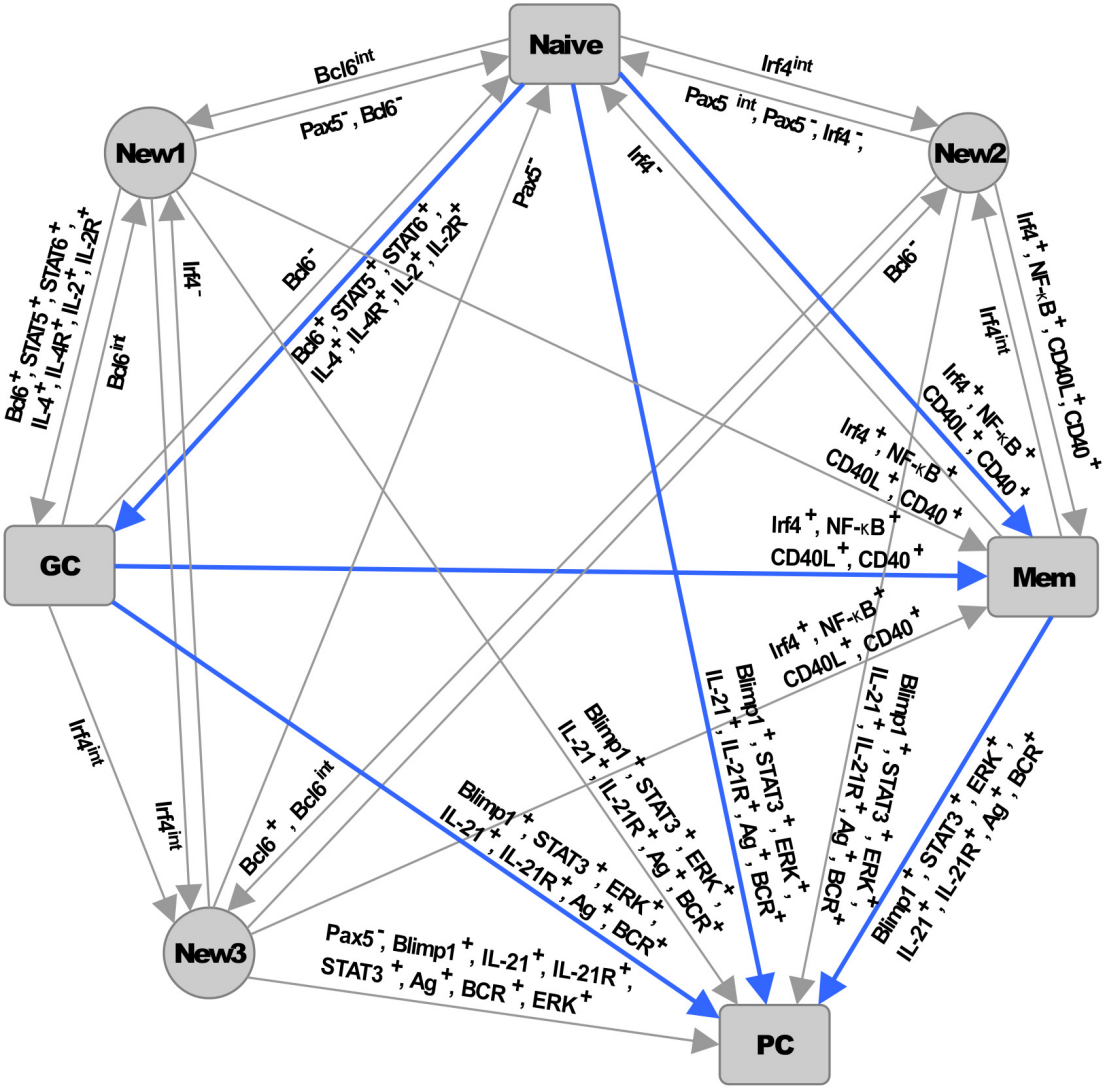
Complete fate map. Nodes represent the fixed point attractors, and the edges correspond to all the possible single-node perturbations able to move the system from one attractor to another. For the continuous model, perturbations are simulated by temporarily change the value of a single node to 0, 1 or 0.5, represented by the symbols “−”, “+” and “*int*”, respectively. For example, IL-2^+^ means that a temporal activation of IL-2 is able to cause the system to move from the Naive attractor to the GC attractor. Biologically relevant differentiation routes are represented as blue arrows.

It has been suggested that progression toward a terminal differentiated state involves several epigenetic changes that reduce the options of a cell to differentiate to other cell types, possibly by several mechanisms that constraint the function of the components of a regulatory network thus reducing the dimensionality of the state space and controlling the compartmentalization of this space into basins of attraction with different sizes [29]. Therefore, the presence of external signals could affect the way the nodes of the network activate in response to these signals which in turn regulate the activation of multiple parts of the network to control the establishment of stationary states of the system and the transitions between these states. Interestingly, no transitions from the PC state to other attractors were obtained in any of the two models, suggesting that the network controls B cell differentiation towards an effector cell fate in an irreversible manner while allowing the transition between precursor cell fates (Fig 5).

### Simulation of mutants

To gain further insight of the dynamical behavior of the B cell regulatory network we systematically simulated all possible single loss- and gain-of-function mutants and evaluated the severity of each mutation by comparing the resulting attractors with those of the wild type model. Loss-of-function mutations were simulated by fixing at 0 the value of a node, whereas gain-of-function was simulated by fixing at 1 the same activation state of a node. For each mutant, its attractors were found, exhaustively in the case of the discrete model, and for the continuous version, by running the dynamical system from 5000 random initial states and solving the equations numerically until the system converged. Tables 3, 4 and 5 shows that the mutants can be grouped according to whether its effect results in the loss of one or more attractors with respect to the wild type model or if it results in the appearance of atypical attractors not found in the wild type model.

**Table 3:** Simulated null mutant attractors.

Mutant model	Obtained pattern	Effect	References
Bach2	[0, 0, 0, 0, 1, 0] Naive-like	Only similar attractors to the wild type fates were found.	[9, 12, 19]
	[0, 1, 0, 0, 1, 0] GC-like		
	[0, 0, 0, 1, 1, 0] Mem-like		
	[0, 0, 1, 1, 0, 1] PC		
Bcl6	[1, 0, 0, 0, 1, 0] Naive	Loss of GC attractor.	[13–15, 57, 58]
	[1, 0, 0, 1, 1, 0] Mem		
	[0, 0, 1, 1, 0, 1] PC		
Blimp1	[1, 0, 0, 0, 1, 0] Naive	Loss of PC attractor. A distinct attractor with high Irf4 levels found.	[54, 56, 59]
	[1, 1, 0, 0, 1, 0] GC		
	[1, 0, 0, 1, 1, 0] Mem		
	[0, 0, 0, 1, 0, 0] Other		
Irf4	[1, 0, 0, 0, 1, 0] Naive	Only Naive and GC attractors are reached by the network. Loss of Mem and PC attractors.	[18, 37, 60, 61]
	[1, 1, 0, 0, 1, 0] GC		
Pax5	[0, 0, 1, 1, 0, 1] PC	Inactivation of Pax5 drives the system to the PC state. An attractor not reported in literature was found.	[8, 30, 62, 63]
	[0, 0, 0, 0, 0, 0] Other		
XBP1	[1, 0, 0, 0, 1, 0] Naive	Mild effect over the PC attractor. Naive, GC and Mem attractors are not affected	[64, 65]
	[1, 1, 0, 0, 1, 0] GC		
	[1, 0, 0, 1, 1, 0] Mem		
	[0, 0, 1, 1, 0, 0] PC-like		

Null mutant attractors. The attractors found for each null mutant model and the literature supporting its effect are summarized, for simplicity, only the patterns of activation for the nodes that conform the core of the network, namely Bach2, Bcl6, Blimp1, Irf4, Pax5 and XBP1 are shown. The steady state pattern for each mutant is shown in the following order: [Bach2, Bcl6, Blimp1, Irf4, Pax5, XBP1].

**Table 4:** Simulated constitutive mutant attractors.

Mutant model	Obtained pattern	Effect	References
Bach2	[1, 0, 0, 0, 1, 0] Naive	Loss of PC attractor. An attractor with active Bach2 and Irf4 was found.	[12]
	[1, 1, 0, 0, 1, 0] GC		
	[1, 0, 0, 1, 1, 0] Mem		
	[1, 0, 0, 1, 0, 0] Other		
Bcl6	[1, 1, 0, 0, 1, 0] GC	Only GC and similar attractor are reached.	[34, 66, 69–71]
	[1, 1, 0, 1, 1, 0] Other		
	[0, 1, 0, 1, 0, 0] Other		
Blimp1	[0, 0, 1, 1, 0, 1] PC	The system stays in the PC state. Loss of Naive, GC and Mem attractors.	[5, 72–76]
Irf4	[1, 0, 0, 1, 1, 0] Mem	The system reaches only the Mem and PC attractors.	[18, 44, 70]
	[0, 0, 1, 1, 0, 1] PC		
Pax5	[1, 0, 0, 0, 1, 0] Naive	Loss of PC attractor, the other three wild type activation patterns are reached by the network.	[63, 67, 77, 78]
	[1, 1, 0, 0, 1, 0] GC		
	[1, 0, 0, 1, 1, 0] Mem		
XBP1	[1, 0, 0, 0, 1, 1] Naive-like	Activation of XBP1 node does not affects the establishment of any of the Naive, GC, Mem or PC attractors. Only similar attractors to the wild type patterns were found.	[54]
	[1, 1, 0, 0, 1, 1] GC-like		
	[1, 0, 0, 1, 1, 1] Mem-like		
	[0, 0, 1, 1, 0, 1] PC		

Constitutive mutant attractors. The attractors found for each mutant model and the literature supporting its effect are summarized, for simplicity, only the patterns of activation for the nodes that conform the core of the network, namely Bach2, Bcl6, Blimp1, Irf4, Pax5 and XBP1 are shown. The steady state pattern for each mutant is shown in the following order: [Bach2, Bcl6, Blimp1, Irf4, Pax5, XBP1].

**Table 5:** Summary of the simulated mutants and external signals.

Mutant models and simulated signals	Resulting attractors with respect to the wild type model
Bach2^+^, Bcl6^+^, Blimp1^−^, Irf4^−^, Pax5^+^	Loss of PC attractor
Bcl6^+^, IL-2^+^, IL-2R^+^, STAT5^+^, IL-4^+^, IL-4R^+^, STAT6^+^, Irf4^+^, CD40L^+^, CD40^+^, NF-*κ*B^+^, Blimp1^+^, Ag^+^, BCR^+^, ERK^+^, IL-21^+^, IL-21R^+^, STAT3^+^	Loss of Naive attractor
Bach2^+^, Bach2^−^, XBP1^+^	Replaced Naive, Mem and GC attractor by similar ones
Bcl6^−^, IL-21^+^, IL-21R^+^, STAT3^+^, Ag^+^, BCR^+^, ERK^+^, CD40L^+^, CD40^+^, NF-*κ*B^+^	Loss of GC attractor
Bcl6^+^, IL-4^+^, IL-4R^+^, STAT6^+^	Only the GC and GC-like attractors are found
Blimp1^−^, Pax5^−^	Atypical attractor found
Blimp1^+^, Ag^+^, BCR^+^, ERK^+^, IL-21^+^, IL-21R^+^, STAT3^+^	Only PC attractor is found
Irf4^+^, CD40L^+^, CD40^+^, NF-*κ*B^+^	Only Mem and PC attractors are found
Irf4^−^	Loss of Mem and PC attractors
Irf4^−^, Ag^+^, BCR^+^, ERK^+^	Loss of Mem attractor
XBP1^+^	Replaced PC attractor by a similar one

Effect of all possible single gain- and loss-of-function mutants of the B cell model with respect to wild type, as reflected by their type of attractors. Symbols “+” and “−” after a node name denote gain-of-function and loss-of-function mutations, respectively. The effect of the continued activation of the nodes pertaining to signaling pathways is also indicated with the symbol “+” and summarized in the table.

Importantly, both the discrete and continuous versions of the model were able to describe most of the reported mutants for the six master regulators that conform the core of the network. For instance, the simulated loss-of-function of the Blimp1 node results in the disappearance of the PC attractor, which is in accordance with the experimentally acknowledged role of Blimp1 as an essential regulator for PC differentiation [54]. Although absence of Blimp1 in B cells impedes PC differentiation, it does not affect the establishment of Naive, GC or Mem cell types [54–56], which is in turn reflected by the model since the network reaches all the Naive, GC and Mem attractors in spite of the loss-of-function of the Blimp1 node (Table 3).

Additionally, for Blimp1 null mutant a distinct attractor was found showing low Pax5 and high Irf4 levels. It has been reported that Pax5 inactivation along with Irf4 induction precedes Blimp1 expression and while Irf4 activation is not sufficient to rescue PC differentiation in the absence of Blimp1, the coordinate expression of both factors is necessary for complete terminal B cell differentiation [56]. Therefore, this attractor may represent a cellular state prior to the PC state.

Similarly to the Blimp1 null mutation, the simulated gain-of-function mutants for the Pax5, Bcl6 or Bach2 nodes also result in the loss of the PC attractor but the other three wild type activation patterns are still reached by the network (Table 4), the constitutive activation of any of these nodes maintains the system in attractors corresponding to precursor B cell fates, in accordance with the observations showing that forced expression of Pax5 or Bach2 in mature B cells inhibit terminal differentiation to PCs and are required to maintain the B cell identity program [12, 66, 67]. Moreover, for the Bach2 gain-of-function model an additional attractor was found. This attractor is characterized by high levels of Bach2 and Irf4 and low Pax5 in a pattern similar to the Mem attractor, this attractor may correspond to a state previous to PC differentiation where Bach2 avoids Blimp1 activation when Pax5 is inactive [56, 68].

The simulated Irf4 loss-of-function results in the loss of PC and Mem cell attractors (See Table 3). Since Irf4 deficient B cells are unable to differentiate into Mem and PCs, the attractors found for this mutant support the role of Irf4 in the formation of PC and Mem cell types [18, 37, 60, 61]. Induction of Irf4 promotes the formation of Mem cells and PC differentiation [18, 44, 70], which is also described by the model as simulated gain-of-function of the Irf4 recovers only two attractors corresponding to the Mem and PC states. Therefore, constitutive activation of the Irf4 node drives the system to the Mem and PC cell fate states.

Conversely, constitutive activation of the Bcl6 node results into three attractors, one of them corresponds to the GC cell pattern, the other two attractors correspond to patterns where Bcl6 is active along with Irf4. These activation patterns coincide with the expression patterns observed for centrocytes, which are Bcl6^+^ Irf4^+^ B cells exiting from the GC reaction [79]. This result suggest that sustained activation of the Bcl6 node drives the system to a GC or GC-like state, in accordance with the reported observations where Bcl6 enforced expression in B cells blocks terminal differentiation and regulates GC formation [34, 66, 71, 80].

Bach2 null mutation does not affects the formation of any of the Naive, GC, Mem or PC cell types, thus confirming its role as a dispensable regulator of B cell terminal differentiation, but a necessary negative regulator for Blimp1 expression and PC formation. Only similar attractors to the wild type fates were found [9, 12, 19].

Bcl6 null mutant mice does not form GC cells but differentiation to Naive, Mem or PC cell types is not affected. Also, Bcl6-deficient B cells can differentiate into Mem cells or PC independently of germinal center reactions. Accordingly the GC attractor is lost in the simulated Bcl6 loss-of-function mutant [13–15, 57, 58].

Deletion of Pax5 in mice results in the loss of B cells from early pro-B stage. Inactivation of Pax5 in mature B cells results in the repression of genes necessary for B cell identity. Pax5 deficient B cells differentiate towards the PC cell fate and show Blimp1 up-regulation. Conditional inactivation of Pax5 in mice mature B cells promotes differentiation toward PCs, in line with the PC attractor found for this mutant. An attractor not reported in literature was found which may correspond to the total loss of expression of the B cell lineage factors [8, 30, 62, 63].

XBP1 is not strictly required for initiation of PC cell differentiation or for previous differentiation stages of terminal B cell differentiation. The network reaches all the wild type attractors [64, 65].

Forced expression of Blimp1 promotes terminal differentiation to PC cells. Only the PC attractor was found for this simulated mutant [5, 72–76].

Loss-of-function of XBP1 affects subsequent PC development but it does not impairs B cell differentiation or the establishment of any of the Naive, GC, Mem and PC cell types [54]. Accordingly, similar attractors to the wild type patterns were found.

It is important to note that not all single loss- or gain-of-function mutants have a severe effect on the dynamics of our B cell differentiation model, since simulated Bach2 and XBP1 constitutive and null mutations result in attractors similar to the wild type, suggesting that these nodes have only a mild effect on the global behavior of the network. However, the Bach2 node is not dispensable since the constitutive activation of this node avoided the network for reach the PC attractor, in accordance with its biological role as an inhibitor of PC differentiation [12]. These results show the contribution of each node to the dynamics of network and therefore indicate the importance of these factors as regulators of the differentiation process.

Given that the expression patterns defining each cell type are controlled by the core module of the regulatory network, the attractors found for the wild-type models as well as for the single loss- and gain-of-function mutants persist even in the absence of external signals. However, as mentioned in the above paragraphs, external stimuli can drive the system from one steady state to another, thus affecting the way the network controls the establishment of different expression patterns. Therefore, we simulated the continuous presence of external signals by fixing the activation value of the nodes representing signaling pathways, namely Ag, BCR, CD40, CD40L, ERK, IL-2, IL-2R, IL-4, IL-4R, IL-21, IL-21R, NF-*κ*B, STAT3, STAT5, and STAT6, in order to analyze how its continued activation influences the behavior of the core regulatory network affecting the appearance and maintenance of multiple cell fates. For clarity, the effect of the continued stimulation by external signals and the effect of the simulated mutants on the stationary patterns reached by the network is summarized in Table 5.

## Discussion

The hematopoietic system is well characterized at the cellular level, and there exist several efforts to reconstruct and analyze parts of its underlying molecular regulatory network to understand the differentiation process of multiple cell types. Network modeling has become an appropriate tool for the systematic study of the dynamical properties of specific regulatory networks and signaling pathways. The dynamic behavior of even relatively simple networks is neither trivial nor intuitive. Moreover, experimental information about the kinetic parameters of the molecules conforming such networks is generally lacking. However, the use of qualitative methods shows that it is possible to predict the existence of expression patterns or pointing at missing regulatory interactions.

The model presented in this work describes the activation states observed experimentally for Naive, GC, Mem and PC cell types. This model is also able to describe the differentiation pattern from Naive B cells to GC, Mem and PC subsets in response to specific external signals. Despite the lack of qualitative information it was possible to reconstruct the regulatory network of B cells and propose a basic regulatory architecture. This model propose the existence of some missing regulatory interactions and activation states not documented in the literature that might play an important role in the context of terminal B cell differentiation. Importantly, these interactions constitute specific predictions that can be tested experimentally. It is also relevant to stress that the proposed regulatory interactions might be attained by way of intermediary molecules not included in the regulatory network. This is so because the whole network modeling approach is based upon the net effect of one node over another, focusing on whether the flow of information is known, rather than relying on the direct physical contact between molecules. Furthermore, the results suggest that the dynamical behavior of the B cell regulatory network is to a large extent determined by the structure of the network rather than the detail of the kinetic parameters, in accordance to analyses of related models [42, 81, 82].

While Boolean networks constitute a valuable modeling approach of choice whenever there is only qualitative data available, for this biological system we wanted to incorporate qualitative continue variables that in addition to the identification of the stationary states as in the discrete model, allows for the analysis of the effect of gradients of external signals. The dynamical behavior of the model resembles the qualitative behavior of the differentiation process by recovering the transition of the system from a Naive state to the terminally differentiated PC state under the presence of external signals. This result recapitulates the directional and branched nature of B cell differentiation events and supports the key role of extracellular signals in the maintenance and instruction of the differentiation process. Importantly, the model allows the exploration of system transitions that describe the differentiation form one cell type to another, it is interesting to note that no transitions from the PC state to other attractors were obtained, suggesting that the B cell regulatory network assures the differentiation towards an effector cell fate in an irreversible manner whereas allowing plasticity of the precursor cell fates.

There are several ways in which our model could be improved in future versions. One general change may be the implementation of the model as a stochastic dynamical system. Although both the stochastic and deterministic models retain the same steady states, the implementation as a stochastic system could be useful to generate information about the probability of the cells to transit from one state to another.

Another possible route of refinement of the models would be the inclusion of a specific time scale. Both the discrete and continuous models presented here use qualitative modeling frameworks, with results having arbitrary time units. In order to incorporate phenomena with specific timescales, it will be necessary either to calibrate the continuous dynamical system by scanning for appropriate values for the parameters, or alternatively make use of a quantitative modeling framework. Also, it possible to add other layers of regulation to the model, for example by incorporating the effect of chromatin remodeling on the availability of some genes. However, given that we were able to recover with a small qualitative network the basic patterns of activation, it is possible that the role played by the levels of regulation not included in the present model may significantly reduce the number of possible transitory trajectories of the system, instead of determining nature and number of the stationary states themselves.

Finally, despite the qualitative nature of the model presented here, we believe it might be used as seed to analyze important biological and clinical phenomena, given that deregulation of the master regulators included in the network are known to be involved in oncogenic events occurring in multiple lymphomas. For instance, aberrant expression of Bcl6 may lead to constitutive repression of genes necessary for exit of the GC program and normal differentiation, therefore contributing to lymphomagenesis [83]. In addition, activation of Irf4 leads to extensive cell proliferation and survival [84]. The present model could serve as a starting framework to test different hypothesis regarding the possible routes by which the expression of the aforementioned factors and other components of the network could be regulated in order to find therapeutic intervention strategies or to test how deregulation of the known mechanisms could lead to pathological conditions, thus contributing to our knowledge on the development of lymphomas.

## Materials and Methods

### Molecular basis of the B cell regulatory network

We inferred the regulatory network controlling terminal B cell differentiation from experimental data available in literature. The evidence used to recover the nodes and interactions of the B cell regulatory network (Fig 2) is summarized in the following paragraphs. The transition from Naive B cells to GC, Mem, and antibody-secreting PCs is regulated by the coordinated activity of transcription factors that act as key regulators of the differentiation process. These factors appear to regulate mutually antagonistic genetic programs and can be divided into those that promote and maintain the B cell program, such as Pax5, Bcl6, and Bach2, and those that control terminal differentiation into memory cells or plasma cells, such as Irf4 and Blimp1 and XBP1 [7].

Pax5 functions as the master regulator of B cell identity, it is expressed at the onset of B cell differentiation and is maintained in all developmental stages of B cells upon commitment to plasma cells. Pax5-deficiency results in the loss of B cell identity and the acquisition of multilineage potential [10]. Pax5 directly inhibits Blimp1 transcription by binding to the promoter of *Prdm1* the gene encoding Blimp1 [11]. In turn, Blimp1 represses Pax5 [78], thus conforming a mutually exclusive regulatory circuit. Along with Pax5, Bach2 avoids PC differentiation and promotes class switch recombination by repressing Blimp1 through binding to a regulatory element on the *Prdm1* gene [12]. Bach2 is positively regulated by Pax5 [31], while being repressed by Blimp1 in PCs, thus creating a mutual inhibition feedback loop [19].

Bcl6 expression is induced upon arrival of Naive B cells into the germinal centers. Bcl6 is a transcription factor essential for germinal center formation, since deficiency of Bcl6 results in the absence of germinal centers in mice [14, 15]. The signals that promote high Bcl6 expression in GC cells are not fully understood. However, it has been shown that mutations that disrupt a negative autoregulatory circuit of Bcl6 deregulate its expression and promote the proliferation of GC cells in dense large B cell lymphomas (DLBCL) [34]. Moreover, it has been reported that there exists a positive regulatory mechanism controlling high Bcl6 expression during the GC phase that overcome its negative autoregulation [35, 36]. In accordance with these data, we found necessary to include in our model a positive autoregulatory interaction for Bcl6 (Bcl6 → Bcl6) in order to account for the required signals that maintain high Bcl6 activation levels in GC cells.

Additionally, the presence of IL-2 and IL-4 produced by follicular T helper cells play an important role in the transition from Naive to GC cells, as these signals are required for the maintenance and proliferation of GC cells. IL-2/IL-2R and IL-4/IL-4R signals are transduced by STAT5 and STAT6, respectively, thus positively regulating the expression of Bcl6 [46]. Bcl6 binds directly to the *Prdm1* promoter and down-regulates the expression of Blimp1 in GC cells, thus preventing the terminal differentiation to PCs [85]. Conversely, Bcl6 is a direct target of Blimp1. This creates a mutual inhibition circuit among Bcl6 and Blimp1 [86]. Maturation of GC cells towards the Mem or PC cell fates requires the downregulation of Bcl6 [17]. This process also depends on the activation of BCR by Ag recognition, as well as on the direct contact of B cells with T helper cells which leads to BCR activation and the proteosomal degradation of Bcl6, mediated by ERK [49].

The direct contact between B and T cells is mediated by the union of CD40 with its ligand CD40L, which in turn activates NF-*κ*B, a positive regulator of Irf4 [16]. Irf4 is a key regulator required for the development of Mem cells from Naive and GC cells, and is involved in the control of CSR and PC differentiation [18, 37]. It has been shown that low levels of Irf4 promote CSR while high Irf4 levels promote PC differentiation. Irf4 inhibits Bcl6 by binding to a regulatory site in the *Bcl6* gene promoter in response to the direct contact of B and T cells [16]. Conversely, Bcl6 is a direct negative regulator of Irf4 in GC cells [87, 88], thus generating a mutual inhibition circuit between Bcl6 and Irf4. Moreover, high Irf4 expression is maintained through direct binding of Irf4 to its own promoter creating a positive autoregulatory circuit [61].

Irf4 also plays an important role in early stages of B cell development where it regulates Pax5 expression through the formation of molecular complexes in the *Pax5* enhancer region [89]. Similarly, Pax5 activation during B cell development is maintained by the transcription factor Ebf1 [33] which in turn is activated by Pax5 [32], therefore conforming a mutually positive regulatory circuit. However, the role of the regulatory circuits between Pax5, Irf4 and Ebf1 during terminal B cell differentiation is not clearly understood. Nevertheless, we found necessary to include these interactions in our model (Pax5 → Pax5 and Irf4 ⊣ Pax5) in order to account for the known activation patterns for these two regulators. Therefore, these interactions constitute predictions of the model that may support an important role of these regulatory interactions during the late stages of B cell differentiation.

The processes of CSR and SHM are controlled by the action of AID [90] which is regulated by the direct binding of Pax5, NF-*κ*B and STAT6 to its regulatory regions in response to IL-4 and CD40 signals [91–93]. AID expression is inhibited in PCs by Blimp1 [5].

Finally, PC differentiation program is regulated by the coordinated activity of Blimp1, Irf4 and XBP1. Blimp1 is specifically expressed in PCs and its activation is sufficient to drive mature B cell differentiation towards the PC fate [56]. Blimp1 is induced by the direct binding of Irf4 to an intronic region of the *Prdm1* gene [18, 61]. Also Blimp1 is involved in Irf4 activation conforming a double positive regulatory circuit. Deficient B cells do not express Irf4 and fail to differentiate into PCs [94, 95]. In turn, Blimp1 activates XBP1 [64] which is normally repressed by Pax5 in mature B cells [65].

### The regulatory network as a discrete dynamical system

Boolean networks constitute the simplest approach to modeling the dynamics of regulatory networks. A Boolean network consists of a set of nodes, each of which may attain only one of two states: 0 if the node is OFF, or 1 if the node is ON [96, 97]. The level of activation for the *i*-th node is represented by a discrete variable *x*_*i*_, which is updated at discrete time steps according to a Boolean function *F*_*i*_ such that *x*_*i*_(*t*+1) = *F*_*i*_[*x*_1_(*t*), *x*_2_(*t*), …, *x*_*n*_(*t*)], where [*x*_1_(*t*), *x*_2_(*t*), …, *x*_*n*_(*t*)] is the activation state of the regulators of the node *x*_*i*_ at time *t*. The Boolean function *F*_*i*_ is expressed using the logic operators ∧ (*AND*), ∨ (*OR*), and ¬ (*NOT*). In our model, all *F*_*i*_s are updated simultaneously, which is known as the synchronous approach. The resulting set of *F*_*i*_s is shown in Table 6.

**Table 6:** Logical rules. The set of Boolean rules defining the regulatory network of the terminal differentiation of B cells.

Logic rule	Description	References
AID ← (STAT6 ∨ (NF-*κ*B ∧ Pax5)) ∧¬ Blimp1	AID node is positively regulated by the presence of Pax5 in response to CD40 and IL-4 signals, transduced by NF-*κ*B and STAT6 respectively. AID is active only if its inhibitor Blimp1 is absent.	[91–93]
Bach2 ← Pax5 ∧¬ Blimp1	Bach2 node is activated if its positive regulator Pax5 is active and the suppressor Blimp1 is absent.	[19, 31]
Bcl6 ← (STAT5 ∨ STAT6 ∨ (Pax5 ∧Bcl6)) ∧¬ (Blimp1 ∨ Irf4 ∨ ERK)	The node Bcl6 is induced in response to IL-2 and IL-4, transduced by STAT5 and STAT6 respectively. Its activation depends on the presence of Pax5 (proposed as a positive interaction), and on the mechanisms maintaining its own expression (proposed as a positive autoregulation). Bcl6 node is repressed if either the nodes Blimp1, Irf4 or ERK are active.	[35, 36, 46]
BCR ← Ag	BCR node is activated by the input node Ag, simulating the presence of extracellular antigen.	[100]
Blimp1 ← (ERK ∨ STAT3) ∨ (Irf4 ∧¬ (Pax5 ∨ Bcl6 ∨ Bach2))	Blimp1 is activated by Irf4 if all its inhibitors, Pax5, Bcl6 and Bach2 are inactive. Blimp1 is induced by Ag and IL-21 which are transduced by ERK and STAT3, respectively.	[49, 50, 52–54]
CD40 ← CD40L	The CD40 node is activated by the input node CD40L simulating the direct contact of B with T cells mediated by the union of the CD40 receptor with its ligand.	[16]
ERK ← BCR	BCR cross-linking promotes ERK activation after Ag stimulation	[49, 51, 76]
IL-2R ← IL-2	The IL-2R node is induced by the input node IL-2, simulating the activation of the IL-2R receptor by IL-2 stimulation, a signal involved in GC differentiation	[4]
IL-4R ← IL-4	The IL-4 input node induces the IL-4R node simulating the activation of the IL-4R receptor activation by the cytokine IL-4 required for GC differentiation.	[4]
IL-21R ← IL-21	The IL-21R receptor is induced by IL-21, a signal required for differentiation toward PCs	[101–103]
Irf4 ← (NF-*κ*B ∨ Irf4) ∨ (Blimp1 ∧¬ Bcl6)	Irf4 is induced in response to CD40L signals, transduced by the node NF-*κ*B. Irf4 regulates its own activation and is positively regulated by Blimp1 if its inhibitor Bcl6 is off.	[16, 18, 61, 89]
NF-*κ*B ← CD40	Activation of the CD40 receptor promotes the activation of the transcription factor NF-*κ*B in response to CD40L stimulation	[16]
Pax5 ← (Pax5 ∨ ¬ Irf4) ∧¬(Blimp1 ∨ ERK)	Pax5 is maintained active by low levels of Irf4, proposed as a negative interaction, and possibly by a positive regulatory circuit with Ebf1 that plays a key role during early B cell differentiation, included as a positive autoregulatory interaction. Pax5 is inhibited if Blimp1 or ERK are present.	[11, 51, 78]
STAT3 ← IL-21R	IL-21 signals are transduced by STAT3, represented in the model as a positive interaction of the IL-21R receptor with STAT3.	[101–103]
STAT5 ← IL-2R	Activation of the IL-2R receptor by IL-2 induces STAT5 activation	[46]
STAT6 ← IL-4R	Activation of the IL-4R receptor induces STAT6 in response to IL-4 stimulation	[91]
XBP1 ← Blimp1 ∧¬ Pax5	XBP1 is activated by Blimp1 if the suppressor Pax5 is absent	[65]

The rules determining the state of activation of each node as a function of its regulatory inputs are expressed by the use of the logic operators ∧ (AND), ∨ (OR), and ¬ (NOT).

We obtained all the attractors of the Boolean model by testing all possible initial states under a synchronous updating scheme using the R package BoolNet [99]. Moreover, we simulated all possible single loss- and gain-of-function mutants by fixing the value of each node to 0 or 1, respectively.

The complete discrete model is available for testing in The Cell Collective (http://www.thecellcollective.org/) model *B cell differentiation* [98]. Furthermore, the model is available as the accompanying file S1 File (*Bcells_model.xml*) in SBMLqual format.

### The regulatory network as a continuous dynamical system

The B cell regulatory network was converted into a continuous dynamical system by using the standardized qualitative dynamical systems method (SQUAD) [104, 105] with the modification by Sánchez-Corrales and colaborators [42] to include into the equations a version of the regulatory logic rule for each node. This methodology offers two main advantages, first, it allows to construct a qualitative model in spite of the lack of kinetic information, making use only of the regulatory interactions of the network, and second, since the external signals are continuous in nature, this methodology permit to study the response of the network to such signals while at the same time allowing a direct comparison with the Boolean model. Moreover, due to its formulation as a set of ordinary differential equations, it may find additional unstable steady states, cyclic behavior, or attraction basins with respect to Boolean approaches [105].

The SQUAD method approximate a Boolean system with the use of a set of ordinary differential equations, where the activation level of a node is represented by a variable *x*_*i*_ which is normalized in the range [0, 1]. This is a dimensionless variable since it represents the functional activation level of a node, but it may be used to represent the normalized concentration of the active form of a molecule or a macromolecular complex. The change of the *x*_*i*_ node over time is controlled by an activation term and a decay term as described by:

$$\begin{array}{c} \frac{dx_{i}}{dt}=\frac{-e^{0.5h_{i}}+e^{-h_{i}\left(\omega_{i}-0.5\right)}}{\left(1-e^{0.5h_{i}}\right)\left(1+e^{-h_{i}\left(\omega_{i}-0.5\right)}\right)}-\gamma_{i}x_{i} \end{array}$$ (1)

In Eq (1) parameters *h*_*i*_ and *γ*_*i*_ are the gain of the input of the node and the decaying rate, respectively. The term *ω*_*i*_ is the continuous form of the logical rule describing the response of the node *x*_*i*_ to its regulatory inputs, as defined for the discrete dynamical system in the previous section. The logical statements defined for the discrete model are converted into their continuous equivalent by changing *A* ∧ *B*, *A* ∨ *B*, and ¬*A* in an expression of classic logic into min(*A*, *B*), max(*A*, *B*), and 1−*A*, respectively, thus creating a fuzzy-logic expression. Note that the term *ω*_*i*_ cannot be applied to all nodes of Fig 2, because there are five of them that do not have any regulatory inputs, therefore equations representing these nodes contain only the term for the decaying rate.

**Fig 6:**
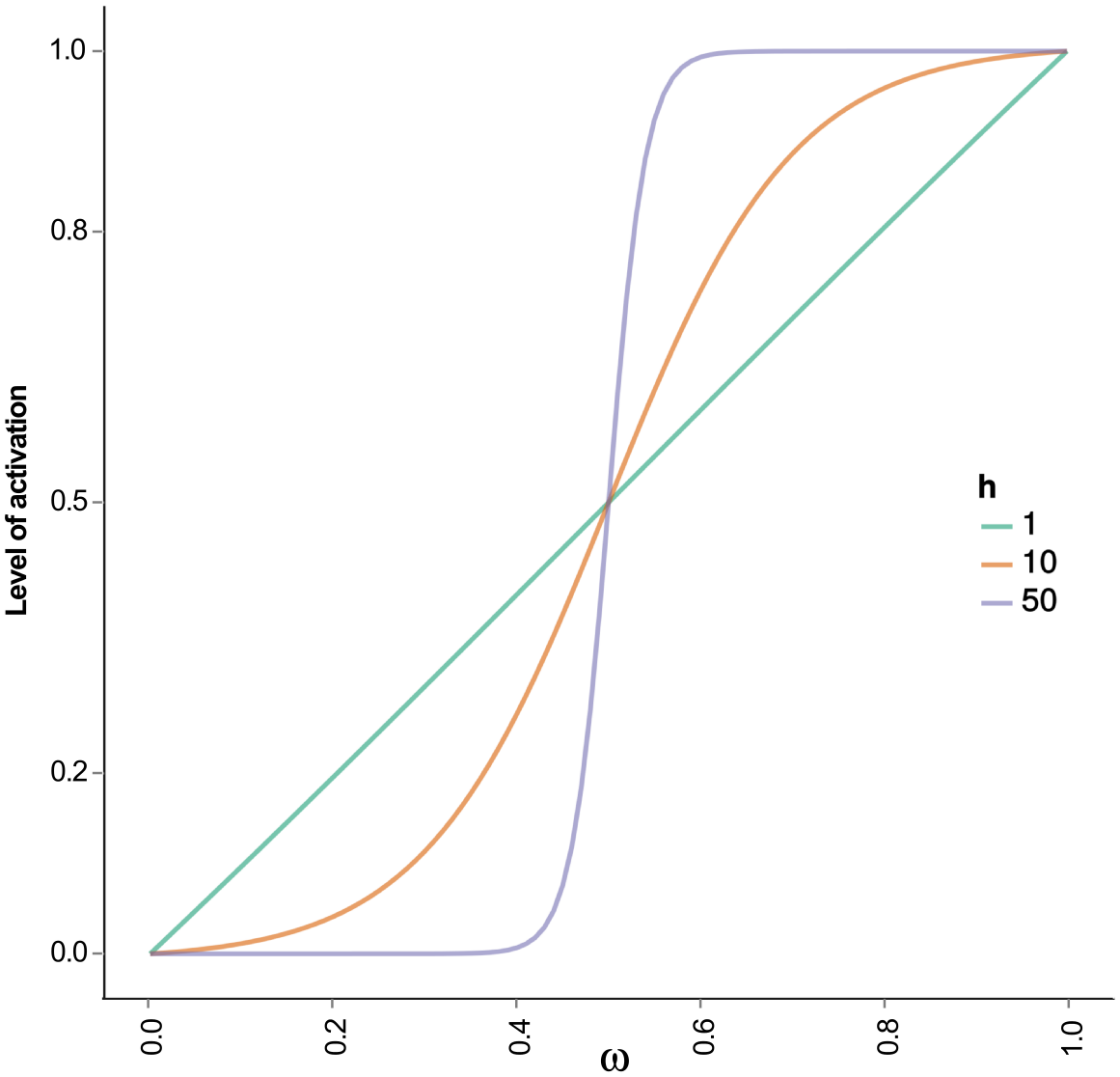
The activation part of Eq (1) is a sigmoid function of the total input of the node (*ω*_*i*_) Regardless of the value of *h*, the sigmoid touches the points (0,0), (0.5,0.5) and (1,1). For values of *h* ≥ 50 the curve resembles a step function.

The activation term for Eq (1) has the form of a sigmoid as a function of the total input to a node *ω*_*i*_, and was constructed so as to pass through the points (0,0), (0.5,0.5), (1,1) for any positive value of *h*. We found that for values of *h* ≥ 50, the curve is very close to a step function; for intermediate values of *h* the function is similar to a logistic curve and as *h* approaches 0 the function is almost a straight line (Fig 6). This characteristic allows the study of different qualitative response curves on the overall behavior of the regulatory network, while at the same time conserving the direct comparison against a Boolean model due to the three fixed points mentioned above. Since there is a lack of published quantitative data that could be used to estimate the values of either of the *h*_*i*_ and *γ*_*i*_ parameters to solve the system of equations, we decided to use a set of default values. Therefore, all *h*’s were set to 50 and *γ* = 1 so as to obtain steep response curves, thus making an easy comparison of the discrete model against the current continuous model and/or forthcoming models.

We found that values *h* ≠ {4,8} and *γ* = 1 recover the experimentally observed patterns of expression S2 Fig. In contrast to the relative insensitivity of changes in the strength of interactions *h*, the attractors are highly sensitive to changes in values of the decay rate *γ*. Eq (1) is constructed in such a way that *γ* has to have a value equal to 1 in order for *x*_*i*_’s to lie in the closed interval [0, 1]. Now, values of *γ* different than 1 make all attractors to disappear S3 Fig. The attractors of the B cell regulatory network model, therefore, are highly dependent on the value used in the parameter specifying the decaying rate.

The resulting dynamical system in shown as S2 Table in the Supporting Information, and available as the supplementary S1 File. Due to the high non-linearity of the continuous system of equations, we located the steady states of this model by numerically solving the system of equations from 500,000 random initial states and letting it converge, with the use of the R package deSolve [106], the detailed attractors found for both the wild type and the mutant models are shown in S2 File.

## Supporting Information

S1 Table Table of interactions. Key references supporting network interactions.
(PDF)

S2 Table The B cell network as a continuous dynamical system. The set of ordinary differential equations conforming the continuous version of the B cell regulatory network model.
(PDF)

S1 Fig Complete fate map for the discrete model. (TIFF)

S2 Fig Location of the fixed-point attractors as a function of the parameter *h*. (TIFF)

S3 Fig Location of the fixed-point attractors as a function of the parameter *γ*. (TIFF)

S1 File SBMLqual format version of the B cell model. Complete model for testing in the The Cell Collective platform (http://www.thecellcollective.org/), model *B cell differentiation* and file Bcell_model.xml.
(XML)

S2 File Detailed attractors of wild type and simulated mutants. (XLSX)
