# Supplementary material for: A Network Model to Describe the Terminal Differentiation of B Cells
Source: PLoS Comput Biol. 2016 Jan 11;12(1):e1004696. doi: 10.1371/journal.pcbi.1004696 (PMC4720151; doi:10.1371/journal.pcbi.1004696)
Supplement: S1 Table — Key references supporting network interactions. (PDF) [file pcbi.1004696.s001.pdf]

S1 Table

Table of interactions Key references supporting network interactions

Interaction	References
Ag → BCR	[1]
Bach2 ⊣ Blimp1	[2–4]
Bcl6 → Bcl6	Predicted in this work
Bcl6 ⊣ Blimp1	[5–8]
Bcl6 ⊣ Irf4	[9–12]
BCR → ERK	[13–15]
Blimp1 ⊣ AID	[16]
Blimp1 ⊣ Bach2	[4]
Blimp1 ⊣ Bcl6	[16–18]
Blimp1 → Irf4	[18, 19]
Blimp1 ⊣ Pax5	[16, 20, 21]
Blimp1 → XBP1	[22–24]
CD40 → NFκB	[25, 26]
CD40L → CD40	[26]
ERK ⊣ Bcl6	[13]
ERK → Blimp1	[14]
ERK ⊣ Pax5	[15]
IL-2 → IL-2R	[27–29]
IL-2R → STAT5	[30]
IL-4 → IL-4R	[27–29]
IL-4R → STAT6	[30, 31]
IL-21 → IL-21R	[27–29]
IL-21R → STAT3	[27–29, 32]
Irf4 ⊣ Bcl6	[17, 26]
Irf4 → Blimp1	[32–34]
Irf4 → Irf4	[34]
Irf4 ⊣ Pax5	Predicted in this work
NFκB → AID	[23, 33, 35]
NFκB → Irf4	[26, 33]
Pax5 → AID	[35, 36]
Pax5 → Bach2	[25, 26]
Pax5 → Bcl6	Predicted in this work

S1 Table : Key references supporting the network interactions (Continued).

Interaction	References
Pax5 ⊣ Blimp1	[20, 37, 38]
Pax5 → Pax5	Predicted in this work
Pax5 ⊣ XBP1	[39, 40]
STAT3 → Blimp1	[32, 41]
STAT5 → Bcl6	[30]
STAT6 → AID	[30, 31]
STAT6 → Bcl6	[30, 42]

References

1. Schmidt C, Kim D, Ippolito GC, Naqvi HR, Probst L, Mathur S, et al. Signalling of the BCR is regulated by a lipid rafts-localised transcription factor, Bright. EMBO J. 2009 Mar;28(6):711–24.
2. Ochiai K, Katoh Y, Ikura T, Hoshikawa Y, Noda T, Karasuyama H, et al. Plasmacytic transcription factor Blimp-1 is repressed by Bach2 in B cells. J Biol Chem. 2006 Dec;281(50):38226–34.
3. Ochiai K, Muto A, Tanaka H, Takahashi S, Igarashi K. Regulation of the plasma cell transcription factor Blimp-1 gene by Bach2 and Bcl6. Int Immunol. 2008 Mar;20(3):453–60.
4. Muto A, Ochiai K, Kimura Y, Itoh-Nakadai A, Calame KL, Ikebe D, et al. Bach2 represses plasma cell gene regulatory network in B cells to promote antibody class switch. EMBO J. 2010 Dec;29(23):4048–61.
5. Shaffer AL, Yu X, He Y, Boldrick J, Chan EP, Staudt LM. BCL-6 Represses Genes that Function in Lymphocyte Differentiation, Inflammation, and Cell Cycle Control. Immunity. 2000 Aug;13(2):199–212.
6. Reljic R, Wagner SD, Peakman LJ, Fearon DT. Suppression of Signal Transducer and Activator of Transcription 3-Dependent B Lymphocyte Terminal Differentiation by Bcl-6. J Exp Med. 2000 Dec;192(12):1841–1848.
7. Vasanwala FH, Kusam S, Toney LM, Dent AL. Repression of AP-1 function: a mechanism for the regulation of Blimp-1 expression and B lymphocyte differentiation by the B cell lymphoma-6 protooncogene. J Immunol. 2002 Aug;169(4):1922–9.
8. Tunyaplin C, Shaffer aL, Angelin-Duclos CD, Yu X, Staudt LM, Calame KL. Direct repression of prdm1 by Bcl-6 inhibits plasmacytic differentiation. J Immunol. 2004 Jul;173(2):1158–65.
9. Gupta S, Jiang M, Anthony A, Pernis AB. Lineage-specific modulation of interleukin 4 signaling by interferon regulatory factor 4. J Exp Med. 1999 Dec;190(12):1837–1848.
10. Ci W, Polo JM, Cerchietti L, Shaknovich R, Wang L, Yang SN, et al. The BCL6 transcriptional program features repression of multiple oncogenes in primary B cells and is deregulated in DLBCL. Blood. 2009 May;113(22):5536–48.
11. Basso K, Saito M, Sumazin P, Margolin Aa, Wang K, Lim Wk, et al. Integrated biochemical and computational approach identifies BCL6 direct target genes controlling multiple pathways in normal germinal center B cells. Blood. 2010 Feb;115(5):975–84.
12. Alinikula J, Lassila O. Gene interaction network regulates plasma cell differentiation. Scand J Immunol. 2011 Jun;73(6):512–9.
13. Niu H, Ye BH, Dalla-Favera R. Antigen receptor signaling induces MAP kinase-mediated phosphorylation and degradation of the BCL-6 transcriptionÂ factor. Genes Dev. 1998 Jul;12(13):1953–1961.
14. Yasuda T, Kometani K, Takahashi N, Imai Y, Aiba Y, Kurosaki T. ERKs induce expression of the transcriptional repressor Blimp-1 and subsequent plasma cell differentiation. Sci Signal. 2011 Jan;4(169):ra25.

15. Yasuda T, Hayakawa F, Kurahashi S, Sugimoto K, Minami Y, Tomita A, et al. B cell receptor-ERK1/2 signal cancels PAX5-dependent repression of BLIMP1 through PAX5 phosphorylation: a mechanism of antigen-triggering plasma cell differentiation. J Immunol. 2012 Jun;188(12):6127–34.

16. Shaffer AL, Lin KI, Kuo TC, Yu X, Hurt EM, Rosenwald A, et al. Blimp-1 Orchestrates Plasma Cell Differentiation by Extinguishing the Mature B Cell Gene Expression Program. Immunity. 2002 Jul;17(1):51–62.

17. Kuo TC, Shaffer AL, Haddad J, Choi YS, Staudt LM, Calame K. Repression of BCL-6 is required for the formation of human memory B cells in vitro. J Exp Med. 2007 Apr;204(4):819–30.

18. Sciammas R, Davis MM. Modular nature of Blimp-1 in the regulation of gene expression during B cell maturation. J Immunol. 2004 May;172(9):5427–40.

19. Kallies A, Hasbold J, Tarlinton DM, Dietrich W, Corcoran LM, Hodgkin PD, et al. Plasma cell ontogeny defined by quantitative changes in blimp-1 expression. J Exp Med. 2004 Oct;200(8):967–77.

20. Mora-López F, Reales E, Brieva JA, Campos-Caro A, Dc W, Mora-lo F, et al. Human BSAP and BLIMP1 conform an autoregulatory feedback loop. Blood. 2007 Nov;110(9):3150–7.

21. Lin KI, Angelin-Duclos C, Kuo TC, Calame K. Blimp-1-dependent repression of Pax-5 is required for differentiation of B cells to immunoglobulin M-secreting plasma cells. Mol Cell Biol. 2002 Jul;22(13):4771–80.

22. Shaffer AL, Shapiro-Shelef M, Iwakoshi NN, Lee AH, Qian SB, Zhao H, et al. XBP1, downstream of Blimp-1, expands the secretory apparatus and other organelles, and increases protein synthesis in plasma cell differentiation. Immunity. 2004 Jul;21(1):81–93.

23. Klein U, Casola S, Cattoretti G, Shen Q, Lia M, Mo T, et al. Transcription factor IRF4 controls plasma cell differentiation and class-switch recombination. Nat Immunol. 2006 Jul;7(7):773–82.

24. Teng Y, Takahashi Y, Yamada M, Kurosu T, Koyama T, Miura O, et al. IRF4 negatively regulates proliferation of germinal center B cell-derived Burkitt's lymphoma cell lines and induces differentiation toward plasma cells. Eur J Cell Biol. 2007 Oct;86(10):581–9.

25. Basso K, Klein U, Niu H, Stolovitzky Ga, Tu Y, Califano A, et al. Tracking CD40 signaling during germinal center development. Blood. 2004 Dec;104(13):4088–96.

26. Saito M, Gao J, Basso K, Kitagawa Y, Smith PM, Bhagat G, et al. A signaling pathway mediating downregulation of BCL6 in germinal center B cells is blocked by BCL6 gene alterations in B cell lymphoma. Cancer Cell. 2007 Sep;12(3):280–92.

27. Linterman MA, Beaton L, Yu D, Ramiscal RR, Srivastava M, Hogan JJ, et al. IL-21 acts directly on B cells to regulate Bcl-6 expression and germinal center responses. J Exp Med. 2010 Feb;207(2):353–63.

28. Zotos D, Coquet JM, Zhang Y, Light A, D'Costa K, Kallies A, et al. IL-21 regulates germinal center B cell differentiation and proliferation through a B cell-intrinsic mechanism. J Exp Med. 2010 Feb;207(2):365–78.

29. Rankin AL, MacLeod H, Keegan S, Andreyeva T, Lowe L, Bloom L, et al. IL-21 Receptor Is Critical for the Development of Memory B Cell Responses. J Immunol. 2011 Jan;186(2):667–74.

30. Scheeren Fa, Naspetti M, Diehl S, Schotte R, Nagasawa M, Wijnands E, et al. STAT5 regulates the self-renewal capacity and differentiation of human memory B cells and controls Bcl-6 expression. Nat Immunol. 2005 Mar;6(3):303–13.

31. Dedeoglu F. Induction of activation-induced cytidine deaminase gene expression by IL-4 and CD40 ligation is dependent on STAT6 and NF B. Int Immunol. 2004 Mar;16(3):395–404.

32. Kwon H, Thierry-Mieg D, Thierry-Mieg J, Kim HP, Oh J, Tunyaplin C, et al. Analysis of interleukin-21-induced Prdm1 gene regulation reveals functional cooperation of STAT3 and IRF4 transcription factors. Immunity. 2009 Dec;31(6):941–52.

33. Sciammas R, Shaffer aL, Schatz JH, Zhao H, Staudt LM, Singh H. Graded expression of interferon regulatory factor-4 coordinates isotype switching with plasma cell differentiation. Immunity. 2006 Aug;25(2):225–36.

34. Shaffer AL, Emre NCT, Lamy L, Ngo VN, Wright G, Xiao W, et al. IRF4 addiction in multiple myeloma. Nature. 2008 Jul;454(7201):226–31.

35. Tran TH, Nakata M, Suzuki K, Begum NA, Shinkura R, Fagarasan S, et al. B cell-specific and stimulation-responsive enhancers derepress Aicda by overcoming the effects of silencers. Nat Immunol. 2010 Feb;11(2):148–54.

36. Gonda H, Sugai M, Nambu Y, Katakai T, Agata Y, Mori KJ, et al. The balance between Pax5 and Id2 activities is the key to AID gene expression. J Exp Med. 2003 Nov;198(9):1427–37.

37. Delogu A, Schebesta A, Sun Q, Aschenbrenner K, Perlot T, Busslinger M. Gene repression by Pax5 in B cells is essential for blood cell homeostasis and is reversed in plasma cells. Immunity. 2006 Mar;24(3):269–81.

38. Nera KP, Kohonen P, Narvi E, Peippo A, Mustonen L, Terho P, et al. Loss of Pax5 promotes plasma cell differentiation. Immunity. 2006 Mar;24(3):283–93.

39. Todd DJ, McHeyzer-Williams LJ, Kowal C, Lee AH, Volpe BT, Diamond B, et al. XBP1 governs late events in plasma cell differentiation and is not required for antigen-specific memory B cell development. J Exp Med. 2009 Sep;206(10):2151–9.

40. Reimold AM, Ponath PD, Li YS, Hardy RR, David CS, Strominger JL, et al. Transcription factor B cell lineage-specific activator protein regulates the gene for human X-box binding protein 1. J Exp Med. 1996 Feb;183(2):393–401.

41. Diehl SA, Schmidlin H, Nagasawa M, van Haren SD, Kwakkenbos MJ, Yasuda E, et al. STAT3-mediated up-regulation of BLIMP1 Is coordinated with BCL6 down-regulation to control human plasma cell differentiation. J Immunol. 2008 Apr;180(7):4805–15.

42. Arguni E, Arima M, Tsuruoka N, Sakamoto A, Hatano M, Tokuhisa T. JunD/AP-1 and STAT3 are the major enhancer molecules for high Bcl6 expression in germinal center B cells. Int Immunol. 2006 Jul;18(7):1079–89.
