# Supplementary material for: A Network Model to Describe the Terminal Differentiation of B Cells
Source: PLoS Comput Biol. 2016 Jan 11;12(1):e1004696. doi: 10.1371/journal.pcbi.1004696 (PMC4720151; doi:10.1371/journal.pcbi.1004696)
Supplement: S2 Table — The set of ordinary differential equations conforming the continuous version of the B cell regulatory network model. (PDF) [file pcbi.1004696.s002.pdf]

S2 Table

The B cell network as a continuous dynamical system The set of ordinary differential equations conforming the continuous version of the B cell regulatory network model.

$$\frac{dAID}{dt} = \frac{-e^{25}+e^{-50(\min(max(STAT6,min(NF\kappa B,Pax5)),1-Blimp1)-0.5)}}{(1-e^{25})(1+e^{-50(\min(max(STAT6,min(NF\kappa B,Pax5)),1-Blimp1)-0.5)})} - AID$$

$$\frac{dAg}{dt} = -Ag$$

$$\frac{dBach2}{dt} = \frac{-e^{25}+e^{-50(\min(Pax5,1-Blimp1)-0.5)}}{(1-e^{25})(1+e^{-50(\min(Pax5,1-Blimp1)-0.5)})} - Bach2$$

$$\frac{dBcl6}{dt} = \frac{-e^{25}+e^{-50(\min(\max(STAT5,STAT6,\min(Pax5,Bcl6)),1-(\max(Blimp1,Irf4,ERK)))-0.5)}}{(1-e^{25})(1+e^{-50(\min(\max(STAT5,STAT6,\min(Pax5,Bcl6)),1-(\max(Blimp1,Irf4,ERK)))-0.5)})} - Bcl6$$

$$\frac{dBCR}{dt} = \frac{-e^{25}+e^{-50(Ag-0.5)}}{(1-e^{25})(1+e^{-50(Ag-0.5)})} - BCR$$

$$\frac{dBlimp1}{dt} = \frac{-e^{25}+e^{-50(\max(\max(ERK,STAT3),\min(Irf4,1-(\max(Pax5,Bcl6,Bach2))))-0.5)}}{(1-e^{25})(1+e^{-50(\max(\max(ERK,STAT3),\min(Irf4,1-(\max(Pax5,Bcl6,Bach2))))-0.5)})} - Blimp1$$

$$\frac{dCD40}{dt} = \frac{-e^{25}+e^{-50(CD40L-0.5)}}{(1-e^{25})(1+e^{-50(CD40L-0.5)})} - CD40$$

$$\frac{dCD40L}{dt} = -CD40L$$

$$\frac{dERK}{dt} = \frac{-e^{25}+e^{-50(BCR-0.5)}}{(1-e^{25})(1+e^{-50(BCR-0.5)})} - ERK$$

$$\frac{dIL2}{dt} = -IL2$$

$$\frac{dIL2R}{dt} = \frac{-e^{25}+e^{-50(IL2-0.5)}}{(1-e^{25})(1+e^{-50(IL2-0.5)})} - IL2R$$

$$\frac{dIL4}{dt} = -IL4$$

$$\frac{dIL4R}{dt} = \frac{-e^{25}+e^{-50(IL4-0.5)}}{(1-e^{25})(1+e^{-50(IL4-0.5)})} - IL4R$$

$$\frac{dIL21}{dt} = -IL21$$

$$\frac{dIL21R}{dt} = \frac{-e^{25}+e^{-50(IL21-0.5)}}{(1-e^{25})(1+e^{-50(IL21-0.5)})} - IL21R$$

$$\frac{dIrf4}{dt} = \frac{-e^{25}+e^{-50(\max(\max(NF\kappa B,Irf4),\min(Blimp1,1-Bcl6))-0.5)}}{(1-e^{25})(1+e^{-50(\max(\max(NF\kappa B,Irf4),\min(Blimp1,1-Bcl6))-0.5)})} - Irf4$$

$$\frac{dNF\kappa B}{dt} = \frac{-e^{25}+e^{-50(CD40-0.5)}}{(1-e^{25})(1+e^{-50(CD40-0.5)})} - NF\kappa B$$

$$\frac{dPax5}{dt} = \frac{-e^{25}+e^{-50(\min(max(Pax5,1-Irf4,Bcl6),1-(\max(Blimp1,ERK)))-0.5)}}{(1-e^{25})(1+e^{-50(\min(max(Pax5,1-Irf4,Bcl6),1-(\max(Blimp1,ERK)))-0.5)})} - Pax5$$

$$\frac{dSTAT3}{dt} = \frac{-e^{25}+e^{-50(IL21R-0.5)}}{(1-e^{25})(1+e^{-50(IL21R-0.5)})} - STAT3$$

$$\frac{dSTAT5}{dt} = \frac{-e^{25}+e^{-50(IL2R-0.5)}}{(1-e^{25})(1+e^{-50(IL2R-0.5)})} - STAT5$$

$$\frac{dSTAT6}{dt} = \frac{-e^{25}+e^{-50(IL4R-0.5)}}{(1-e^{25})(1+e^{-50(IL4R-0.5)})} - STAT6$$

$$\frac{dXBP1}{dt} = \frac{-e^{25}+e^{-50(\min(Blimp1,1-Pax5)-0.5)}}{(1-e^{25})(1+e^{-50(\min(Blimp1,1-Pax5)-0.5)})} - XBP1$$
