# Supplementary figures and images for: A Network Model to Describe the Terminal Differentiation of B Cells
Source: PLoS Comput Biol. 2016 Jan 11;12(1):e1004696. doi: 10.1371/journal.pcbi.1004696 (PMC4720151; doi:10.1371/journal.pcbi.1004696)

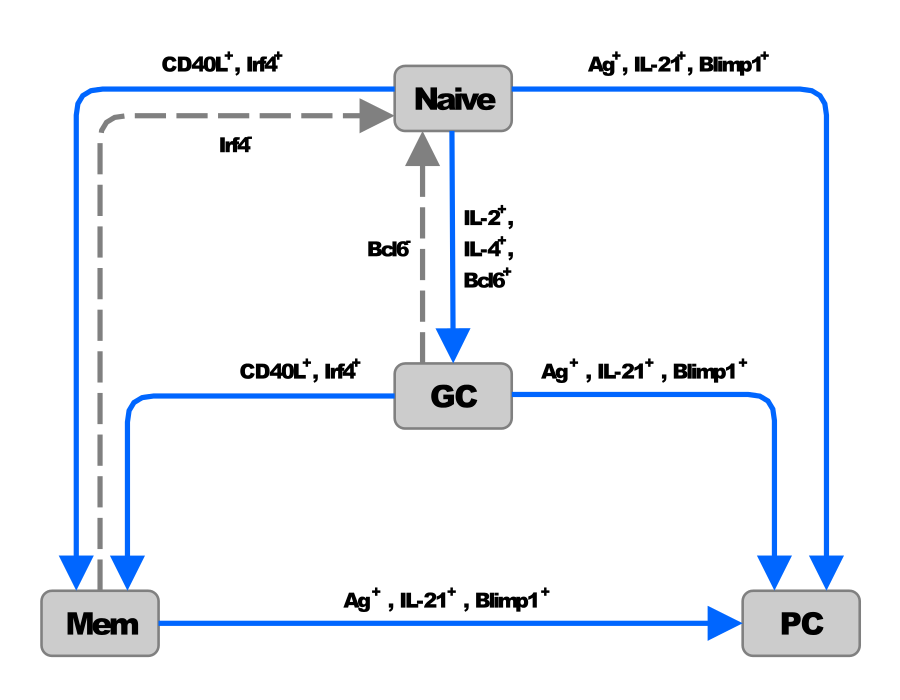

Supplement: S1 Fig — (TIFF) [file pcbi.1004696.s003.tiff]

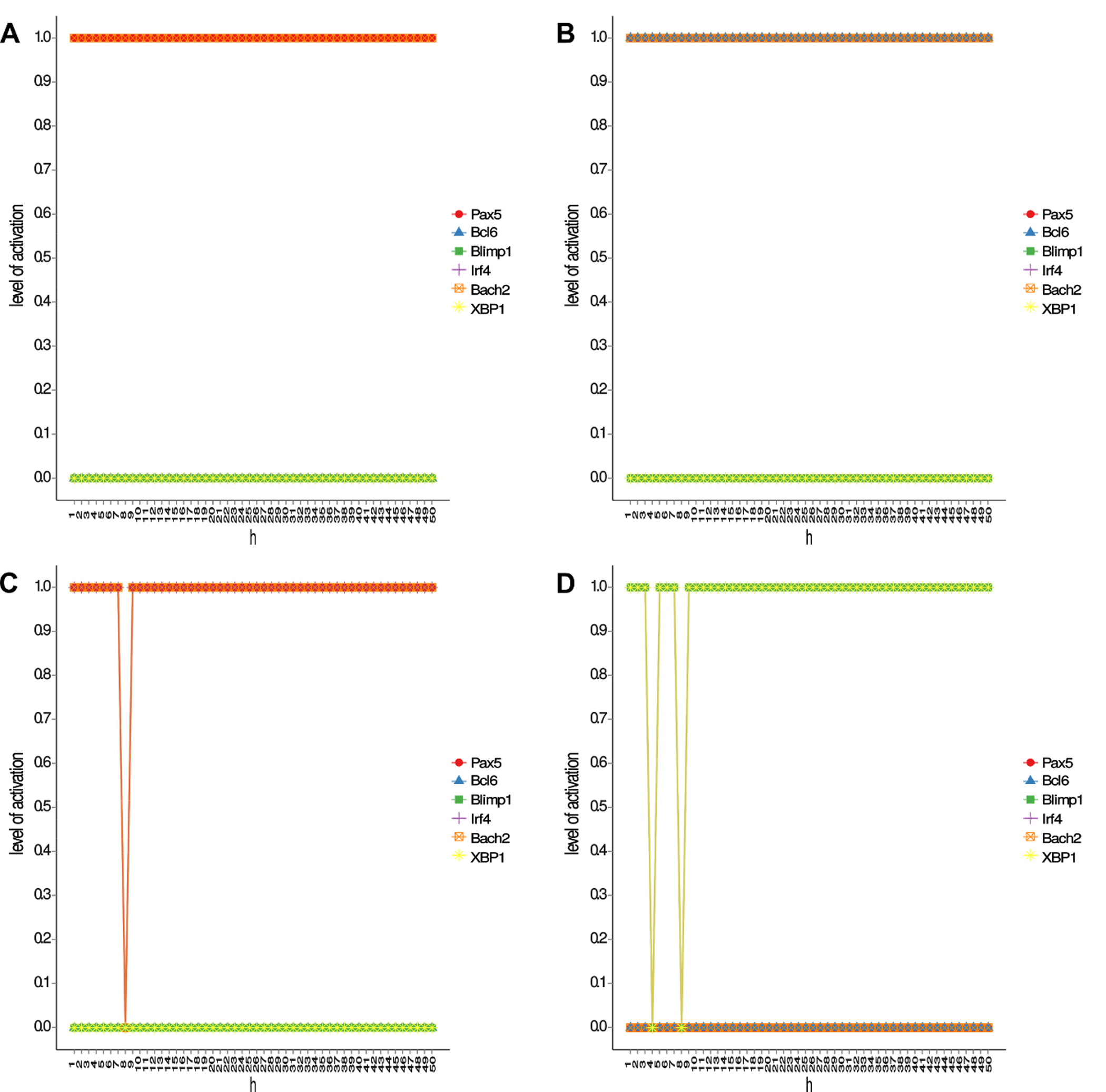

Supplement: S2 Fig — (TIFF) [file pcbi.1004696.s004.tiff]

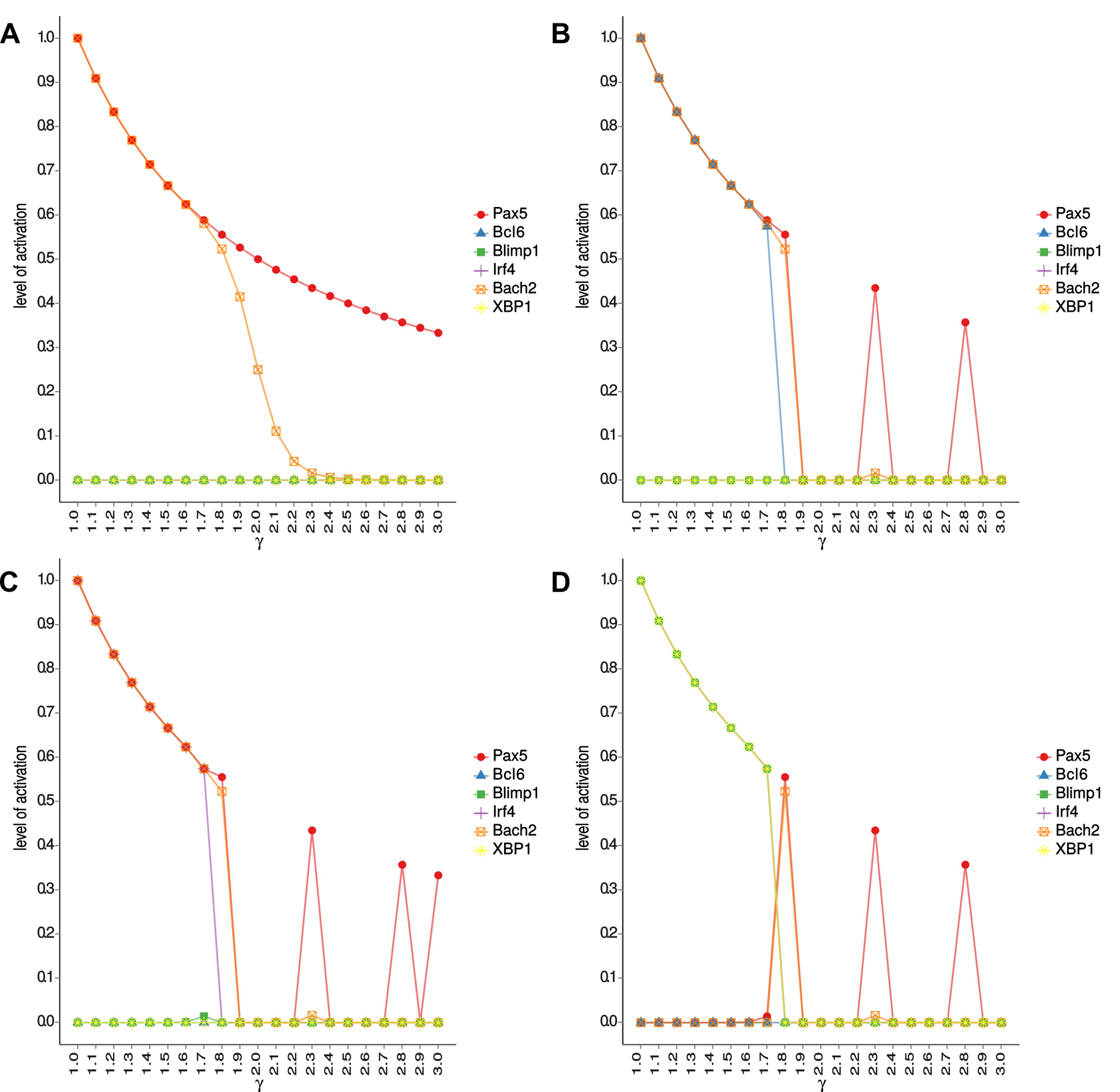

Supplement: S3 Fig — (TIFF) [file pcbi.1004696.s005.tiff]
